# Treatment of liver cirrhosis using hepatocyte-derived liver progenitor-like cells: a prospective, open-label, single-arm, safety trial

**DOI:** 10.1038/s41421-025-00831-y

**Published:** 2025-11-05

**Authors:** Kang He, Xue-Jing Zhu, Yao-Ping Shi, Wei-Jian Huang, Tai-Hua Yang, Zhi-Feng Xi, Qi-Gen Li, Han-Yong Sun, Li-Jun Qian, Xiao-Song Chen, Pei-Ying Li, Xu Zhou, Gui-Ying Gu, Fan Li, Wen-Ming Liu, Cai-Yang Chen, Jie Zhao, Hong-Ping Wu, Fang-Rong Yan, Michael Ott, Amar Deep Sharma, Hui Liu, Wei-Feng Yu, Bo Zhai, He-Xin Yan, Qiang Xia

**Affiliations:** 1 Department of Liver Surgery, Renji Hospital, School of Medicine, Shanghai Jiao Tong University, Shanghai, China; 2 Celliver Biotechnology Inc., Shanghai, China; 3 Department of Interventional Oncology, Renji Hospital, School of Medicine, Jiao Tong University, Shanghai, China; 4 Department of Anesthesiology and Critical Care Medicine, Renji Hospital, School of Medicine, Shanghai Jiao Tong University, Shanghai, China; 5 Key Laboratory of Anesthesiology (Shanghai Jiao Tong University), Ministry of Education, Shanghai, China; 6 Department of Radiology, Renji Hospital, School of Medicine, Shanghai Jiao Tong University, Shanghai, China; 7 Department of Infectious Diseases, Renji Hospital, School of Medicine, Shanghai Jiao Tong University, Shanghai, China; 8 Department of Clinical Laboratory, Eastern Hepatobiliary Surgery Hospital, Naval Medical University, Shanghai, China; 9 Department of Biostatistics, China Pharmaceutical University, Nanjing, Jiangsu China; 10 Department of Gastroenterology, Hepatology and Endocrinology, Hannover Medical School, Hannover, Germany; 11 Department of Hepatic Surgery, Eastern Hepatobiliary Surgery Hospital, Naval Medical University, Shanghai, China

**Keywords:** Regeneration, Reprogramming

## Abstract

Liver transplantation remains constrained by the scarcity of donor organs and the risks inherent in the procedure, underscoring the urgent need for novel cirrhosis therapies. We developed a protocol to convert human primary hepatocytes into expandable hepatocyte-derived liver progenitor-like cells (HepLPCs), which secrete high levels of matrix metalloproteinases and hepatocyte growth factor. In a thioacetamide-induced rat model of cirrhosis, human HepLPCs demonstrated potent anti-fibrotic properties and promoted liver regeneration. Biodistribution studies revealed that most xenogenic HepLPCs were cleared from the body within one week, suggesting that their therapeutic benefits likely arise from paracrine signaling rather than long-term engraftment. We initiated a first-in-human clinical trial involving nine patients with cirrhosis to evaluate the feasibility and safety of HepLPCs. Preclinical toxicity assessments in 36 crab-eating macaques confirmed the safety of HepLPC treatment. In the clinical trial, nine patients (mean age: 53 years), primarily with HBV-related cirrhosis, received HepLPCs via trans-hepatic arterial infusion without immunosuppressants. No serious adverse event was observed, and the minor adverse events were consistent with those commonly seen in cirrhosis patients. The treatment was well tolerated, with no transfusion reactions or dose-limiting toxicities. While significant changes in Child-Pugh and MELD scores were not observed, some patients showed improvements in liver biochemical parameters, coagulation profiles, and portal hypertension indicators during the six-month follow-up. These findings indicate that HepLPC therapy is safe and feasible, offering a promising new strategy for treating cirrhosis. Further clinical trials are needed to assess its efficacy in patients with decompensated cirrhosis and acute-on-chronic liver failure.

## Introduction

Liver cirrhosis represents a severe medical condition characterized by progressive scarring and liver impairment, leading to the eventual loss of vital functions. It often arises from chronic liver diseases like viral hepatitis, alcoholism, nonalcoholic fatty liver disease, autoimmune liver disorders, or damage from drugs or toxins. Cirrhosis can lead to complications such as portal hypertension, jaundice, liver cancer, and even liver failure^1^.

Although liver transplantation stands as the definitive cure for end-stage liver disease, the demand eclipses the availability of suitable donor organs^2^. Additionally, transplantation poses various risks, including peri-transplant complications, rejections, and post-transplant infections. The scarcity of transplant opportunities and the absence of effective alternatives to reverse cirrhosis underline the persistent unmet medical need for cirrhosis patients.

Fetal hepatocyte transplantation without using immunosuppressants has been explored in clinical settings for managing liver cirrhosis, showing promise as a bridge therapy^3–5^. However, ethical concerns and limitations in the application of fetal hepatocytes necessitate alternative strategies for cell transplantation to enhance liver cirrhosis treatment and stimulate regeneration. Recent studies have revealed the potential of chemically reprogramming mature hepatocytes into proliferative liver progenitor-like cells, termed hepatocyte-derived liver progenitor-like cells (HepLPCs)^6,7^. These cells, sharing gene expression profiles with fetal liver cells, have shown promise, though their impact on liver cirrhosis remains unexplored.

In this study, we successfully converted human primary hepatocytes into expandable HepLPCs through chemical reprogramming. These HepLPCs, cultivated to produce factors known to stimulate liver regeneration and inhibit fibrosis, demonstrated anti-fibrotic effects and regenerative potential in a rat model of liver cirrhosis during pre-clinical investigations. Following a toxicological risk assessment, a clinical study involving nine patients with liver cirrhosis further affirmed the safety and potential efficacy of HepLPCs in treating liver cirrhosis. Our comprehensive findings from pre-clinical and clinical studies underscore the safety and promising therapeutic efficacy of HepLPCs, shedding light on a potential breakthrough in addressing the unmet medical needs of patients with liver cirrhosis.

## Results

### Good manufacturing practice (GMP)-compliant manufacture and quality assurance of HepLPCs

**Fig. 1:**
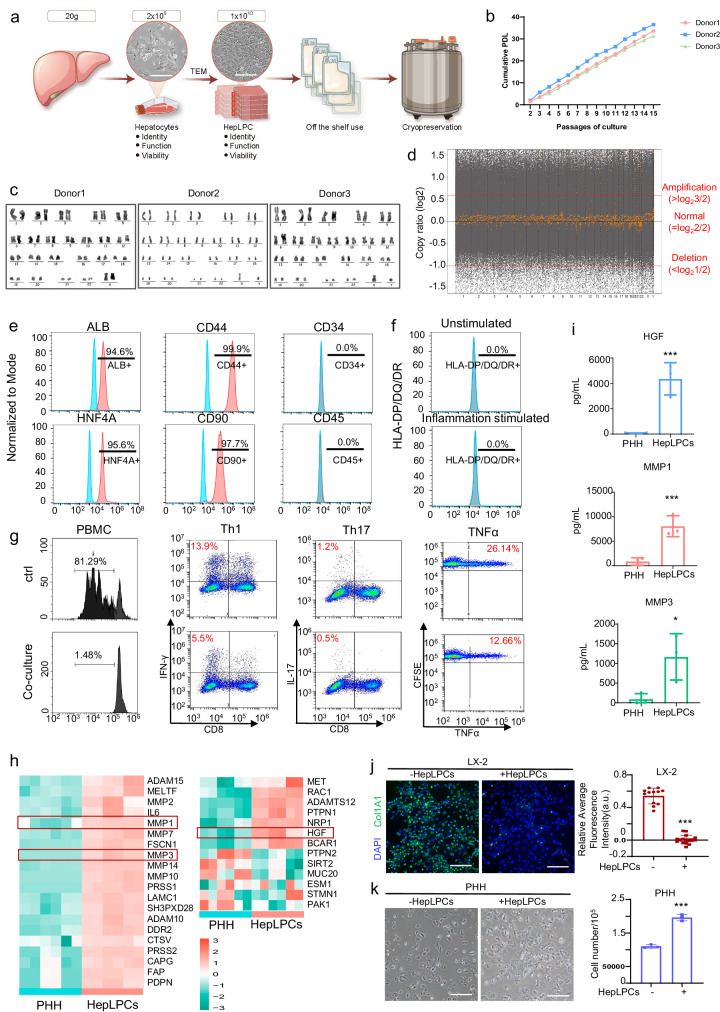
Characterization of GMP-compliant manufacture of HepLPCs. **a** Schematic diagram for the large-scale expansion and cryopreservation of HepLPCs. Light microscopy images of Hepatocytes and HepLPCs cultured in TEM, Scale bars: 200 μm. **b** PDLs calculated for the three donors. **c** Representative karyotype images of three independently established HepLPCs. **d** Copy number variation analysis between HepLPCs and PHH samples. The gray dots represent the log_2_ ratio of the numbers of reads between HepLPCs and PHH samples at the starting positions of each binning window. The yellow dots represent the log2 ratio of the median value ratio at the starting positions of each binning window. The black line represents the log_2_ ratio that equals to 0, which reflects that both samples are completely identical. The red dashed lines indicate the thresholds for determining DNA amplifications/deletions: a log_2_ DNA copy ratio greater than log_2_3/2 is considered as amplification, while a log_2_ DNA copy ratio less than log_2_1/2 is considered as deletion. **e** Flow cytometric analysis showing the proportion of ALB-, HNF4A-, CD44-, CD90-, SOX9-, CD34- and CD45-positive cells in the populations. Red, positive cells; blue, negative controls. **f** HepLPCs were cultured in presence or absence of the cocktail of proinflammatory cytokines. After 24 h, cells were harvested and stained with the antibodies specific for the cell surface markers HLA-DP/DQ/DR. Expression was analyzed by flow cytometry. **g** PHA-stimulated PBMCs were cultured individually or co-cultured with the HepLPCs, and flow cytometry was used to detect the proliferation of PBMCs, subsets of Th1 (CD4^+^/IFN-γ^+^) cells, Th17 (CD4^+^/Th17A^+^) cells, as well as the expression of TNFα. **h** Heatmap showing the gene expression levels associated with the regulation of extracellular matrix disassembly and HGF receptor signaling pathway. *n* = 3 donors (2 independent experiments for each donor). **i** ELISA validation of the HGF, MMP1, and MMP3 secretion levels in HepLPCs and PHHs. *n* = 3 donors (3 independent experiments for each donor). *P < 0.05, **P < 0.01. Data are expressed as mean ± SD, two-tailed Student’s *t*-test. **j** The in vitro experiment used the human hepatic stellate cell line LX2 as target cells, co-culturing the culture supernatant of HepLPCs with LX2 activated by TGF-β1. Immunofluorescence was performed to label and quantify the fibrotic marker Col1A1. Scale bars: 100 μm. **k** Human primary hepatocytes as target cells were cultured with the supernatant from HepLPC culture. Cell counting was used to evaluate the effect of HepLPCs on hepatocyte proliferation. Scale bars: 100 μm.

Under GMP conditions, human primary hepatocytes were successfully converted into expandable HepLPCs within an optimized culture condition containing animal-free growth factors based on transition and expansion medium (TEM) as described previously^7^. Briefly, TEM is composed of DMEM/F12 supplemented with human platelet lysate (HPL) and the following growth factors or small molecules: 20 ng/mL hepatocyte growth factor (HGF), 20 ng/mL EGF, 10 μM Y27632, 3 μM CHIR99021, 1 μM A8301, 1 μM S1P and 5 μM LPA. The process of producing GMP-grade HepLPCs involves meticulous steps to ensure safety, efficacy, and consistency. After rigorous testing of reagents and standard operating procedures, the manufacturing scales up under GMP conditions, establishing a two-tiered banking system with primary and master cell banks. To qualify for clinical use, the HepLPCs were assessed in terms of cell identity, sterility, and safety, thus establishing in-process control and product release criteria (Fig. 1a). This precision allows for the production of HepLPCs that meet pre-defined specifications, enabling the development of an off-the-shelf product. GMP-compliant HepLPCs exhibited a homogeneous epithelial morphology (Fig. 1a), with over 90% viability before and after freezing. The robust and reproducible expansion is evident through multiple hyperflask passages, resulting in three lots with an average population doubling level (PDL) (Fig. 1b). Karyotypic analyses of GMP-compliant HepLPCs lines from 3 donors showed that they could maintain normal diploid karyotypes, even at passage 10 (Fig. 1c). Next, we employed whole genome sequencing^8^ to scrutinize the genomic integrity and stability of HepLPCs. To ensure a relevant and controlled comparison, we conducted a direct analysis with primary hepatocytes (PHHs) obtained from the same donors (Fig. 1d). The analysis revealed that the reprogramming process induced by small molecules did not lead to any significant long-segment deletions or amplifications across the genome. These GMP-compliant HepLPCs expressed liver-specific genes (*ALB* and *HNF4A*) and stem/progenitor genes (*CD44* and *CD90*), as determined by flow cytometry analysis (Fig. 1e). Additionally, these cells did not express the MHC class II antigens HLA-DP, DQ, and DR, indicating their low immunogenicity (Fig. 1e). Furthermore, under inflammatory treatment, the expression of HLA-DR/DP/DQ did not show any significant increase (Fig. 1f). This characteristic offers a considerable advantage, allowing for the use of HepLPCs without the requirement for immunosuppressants^9^. Consistent with these findings, co-culturing PHA-activated PBMCs with HepLPCs significantly suppressed T cell proliferation and the expression of the pro-inflammatory cytokine TNF-α. Moreover, HepLPCs markedly inhibited the Th1 (CD4^+^/IFN-γ^+^) and Th17 (CD4^+^/IL-17A^+^) subsets within CD4^+^ T lymphocytes (Fig. 1g). These immune-regulatory characteristics were shared by mesenchymal stem cells, which confer their safe application clinically without using immunosuppressants.

Next, we conducted bulk RNA sequencing (RNA-seq) analysis on GMP-compliant HepLPCs and their corresponding PHHs (Supplementary Fig. S1). Transcriptomic heatmap analysis revealed HepLPCs display a bipotent progenitor phenotype, evidenced by simultaneous suppression of mature hepatocyte metabolic genes (*CYP3A4*, *UGT1A9*, *FAH*, *ALB*) and enrichment of progenitor/cholangiocyte marker genes (*KRT19*, *EPCAM*, *CCL2*, *SOX9*, *TGFB1*)^10,11^ (Supplementary Fig. S2). Gene Set Variation Analysis (GSVA) revealed that enriched GO-BP and KEGG pathways in HepLPCs were related to cell proliferation and regeneration, including WNT, NOTCH, and Hippo signaling pathways, indicating they retain progenitor cell characteristics (Supplementary Fig. S3).

Notably, HepLPCs were closely associated with the regulation of extracellular matrix disassembly and HGF receptor signaling pathways, with significantly upregulated markers like HGF and matrix metalloproteinases (MMPs) (Fig. 1h; Supplementary Fig. S3a). ELISA analysis showed that HepLPCs secreted significantly higher amounts of HGF, MMP1, and MMP3 compared to PHH (Fig. 1i). HGF is crucial for liver regeneration, while MMPs are involved in extracellular matrix degradation, tissue repair, and wound healing. In vitro experiments confirmed that the HepLPC culture supernatant effectively degraded Col1a1 (an established marker of active fibrogenesis) from activated LX-2 cells, a human hepatic stellate cell line, and promoted human primary hepatocyte proliferation (Fig. 1j, k). These effects could be blocked by general MMPs inhibitors and HGF/c-MET signaling antagonists, respectively (Supplementary Fig. S4). These findings suggest that HepLPCs can enhance liver regeneration and reduce fibrotic responses.

### Improvement of liver injury and cirrhosis by HepLPCs in a rat model

**Fig. 2:**
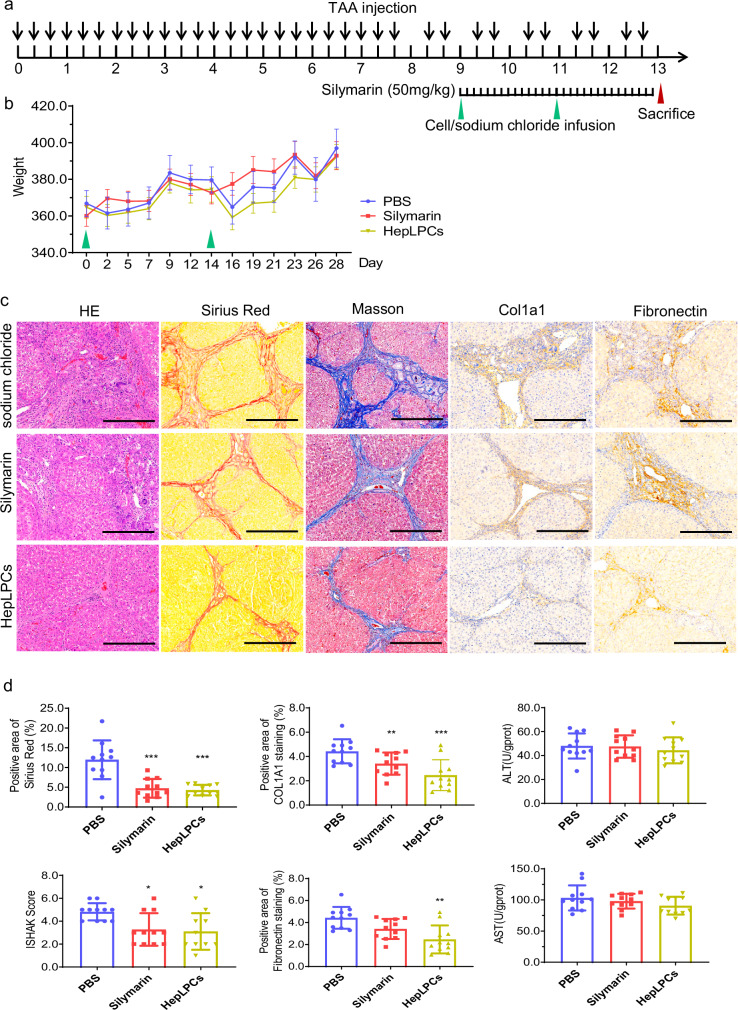
GMP-compliant HepLPCs attenuated rat liver cirrhosis induced by thioacetamide. **a** A schematic overview of the experimental design of the TAA-induced cirrhosis model. **b** The changes of body weight from different groups after treatments. **c** Representative images of H&E stained, Sirius Red stained, Masson’s Trichrome stained, and Col1A1 and fibronectin immuno-stained liver samples. (*n* = 12). Scale bars: 100 μm. **d** Measurement of liver fibrosis score and quantification of Sirius Red staining areas after different treatments. Levels of AST and ALT in rats after different treatments. Quantification of Col1A1- and fibronectin-stained liver samples from different groups. Results are shown as the mean ± SD of three independent experiments. *P < 0.05; **P < 0.005; ***P < 0.001.

To explore the therapeutic potential of human HepLPCs in cirrhosis, we conducted an additional experiment using a thioacetamide (TAA)-induced advanced cirrhosis model in rats to evaluate the therapeutic potential of human HepLPCs transplantation (Fig. 2a). Notably, human HepLPCs demonstrated a lack of expression of MHC class II antigens (HLA-DP, DQ, DR) and exhibited a suppressive effect on T cell proliferation, obviating the need for immunosuppressants during transplantation. The experimental protocol, illustrated in Fig. 2a, involved inducing liver cirrhosis in rats through intraperitoneal administration of TAA (200 mg/kg) three times a week for 13 weeks. After 8 weeks, the rats were divided into three groups. In the HepLPCs groups, rats received transplantation of HepLPCs via portal vein every 2 weeks (twice in total) at 3 × 10⁶ cells/kg (*n* = 12 per group). The positive control group received silymarin (50 mg/kg) daily for 4 weeks to inhibit fibrosis (*n* = 12), while the negative control group received 3 mL/kg of 0.9% sodium chloride every 2 weeks (*n* = 12). Throughout the experiment, the rats in each group exhibited steady weight gain, and there was no reported death following administration or cell transplantation (Fig. 2b).

**Fig. 3:**
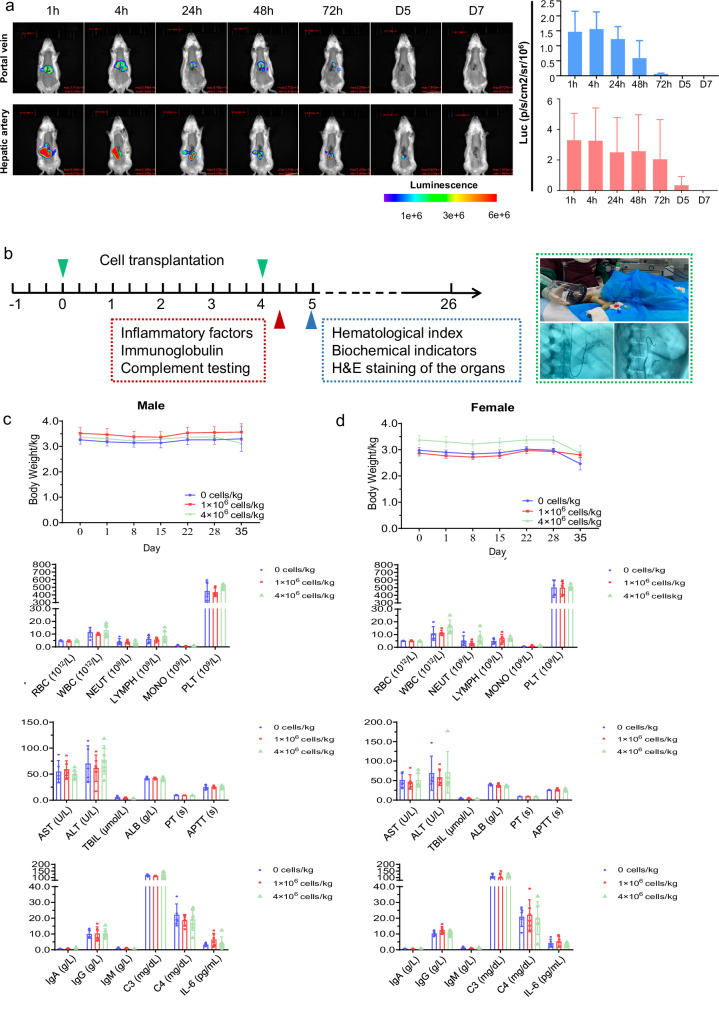
Biodistribution and toxicity of the transplanted HepLPCs. **a** Live images of the GFP/luciferase-labeled HepLPCs in the liver of rats in high-dose groups (4 × 10^6^ cells/kg) at 1 h, 4 h, 24 h, 48 h, 72 h, day5 and day7 post-dosing. Three rats at each time point. Results are shown as the mean ± S.D. **b** Schematic diagram depicting the experimental design for toxicology study of crab-eating macaques. **c** The body weight change, hematological index and biochemical indicators at day 34/35, as well as inflammatory factors, immunoglobulin and complement testing at day 28 in male crab-eating macaques after HepLPCs transplantation (doses of 0, 1 × 10^6^, and 4 × 10^6^ cells/kg, respectively). **d** The body weight change, hematological index and biochemical indicators at day 34/35, as well as inflammatory factors, immunoglobulin and complement testing at day 28 in female crab-eating macaques after HepLPC transplantation (doses of 0, 1 × 10^6^, and 4 × 10^6^ cells/kg, respectively).

As shown in Fig. 2c, the beneficial effects of human HepLPCs, including the reduced accumulation of extracellular matrix and improved liver function, were observed in the TAA-induced model. This therapeutic effect was further substantiated by a significant reduction in the protein levels of Col1a1 and fibronectin by using IHC analysis in the HepLPCs-treated rats, highlighting the therapeutic potential of HepLPCs in mitigating liver fibrosis (Fig. 2c, d). Although changes in serum ALT and AST levels did not reach statistical significance, likely due to the mild elevation of these enzymes in the TAA model as well as the limited persistence of human HepLPCs in rat models (Fig. 3a), the trends suggest a potential ameliorating effect of HepLPCs on liver damage. These findings strongly support the efficacy of HepLPCs therapy in mitigating liver injury and highlight their potential as a therapeutic intervention for liver cirrhosis.

### Biodistribution and toxicological study of transplanted HepLPCs

To monitor the biodistribution and persistence of human HepLPCs in immunocompetent animals, we transplanted GFP/Luciferase-labeled human HepLPCs at a dose of 4 × 10⁶ cells/kg into wild-type SD rats via either the hepatic artery or portal vein. This pharmacokinetic study tracked cell kinetics in the rat livers at various post-infusion time points (Fig. 3a). We observed a consistent decline in HepLPC levels in liver tissues over time, regardless of the transplantation route (Fig. 3a). Notably, HepLPCs introduced via the hepatic artery exhibited longer persistence compared to those delivered through the portal vein, with most cells being cleared within one week. These findings support the idea that human HepLPCs may exhibit immune regulatory effects, as they were able to survive for up to one week, even in wild-type rat models.

To evaluate the safety of HepLPCs transplantation under clinically relevant conditions, we performed repeated-dose toxicity tests in 36 crab-eating macaques, with equal numbers of males and females in each group. Animals received two intrahepatic arterial administrations of HepLPCs (1 × 10⁶ or 4 × 10⁶ cells/kg) at a 4-week interval without immunosuppressive therapy (Fig. 3b). Toxicity was assessed by measuring inflammatory factors, immunoglobulins, and complement on day 28, as well as hematologic toxicity, blood biochemistry, and anatomic pathology on days 34/35 post-transplantation. No significant abnormalities in body weight changes were observed compared to the control groups, and there were no fatalities following HepLPCs administration (Fig. 3c, d). Whole-blood hematology analysis on day 34/35 showed no significant changes, indicating no hematologic toxicity. Blood levels of AST, ALT, total bilirubin (TBIL), and albumin (ALB) did not show statistical differences, and coagulation parameters remained stable. Inflammatory factors, immunoglobulins, and complement levels, including IgA, IgG, IgM, C3, C4, and IL-6, remained stable (Fig. 3c, d). Major organs collected on day 34/35 showed intact liver architecture with no macroscopic tumors (Supplementary Fig. S5). No morphological or histological changes were found in the kidney, testis, intestine, heart, spleen, or lung, reinforcing the safety of HepLPC transplantation.

To clarify the tumorigenic risk of HepLPCs, we conducted a tumorigenicity study in immunodeficient mice. The study employed 24 B-NDG mice (NOD.CB17-Prkdc^scid^ Il2rg^tm1Bcgen^/Bcgen), equally divided into two groups: (1) a positive control group receiving subcutaneous inoculation of 1 × 10⁶ HeLa cells/mouse in the right flank, and (2) a HepLPCs treatment group receiving 1 × 10⁷ HepLPCs/mouse via identical administration route. The day of cell inoculation was designated as day 0, and tumor volumes were measured twice weekly for the first 6 weeks after inoculation and once weekly from week 7 to week 16. By the end of the experiment at day 115, the tumorigenic rate of the positive control group was 100%, while that of the test substance group was 0%, indicating that HepLPCs have no in vivo tumorigenic risk (Supplementary Fig. S6).

### Feasibility and safety of HepLPC treatment in patients with liver cirrhosis

**Fig. 4:**
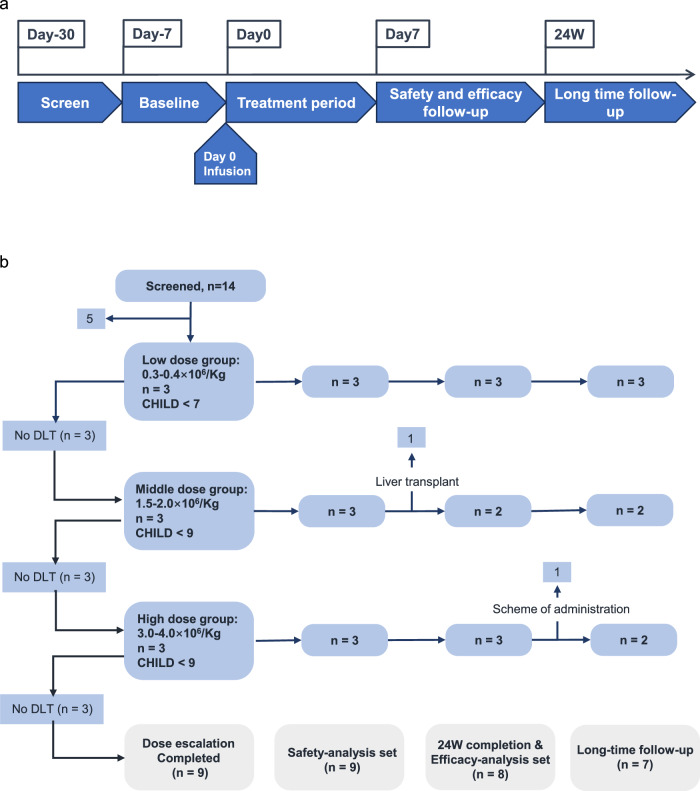
Study design and patient disposition. **a** Schematic representation of the study design in accordance with the modified protocol. **b** Schematic description of patient disposition and allocation to cohorts and analysis sets.

**Fig. 5:**
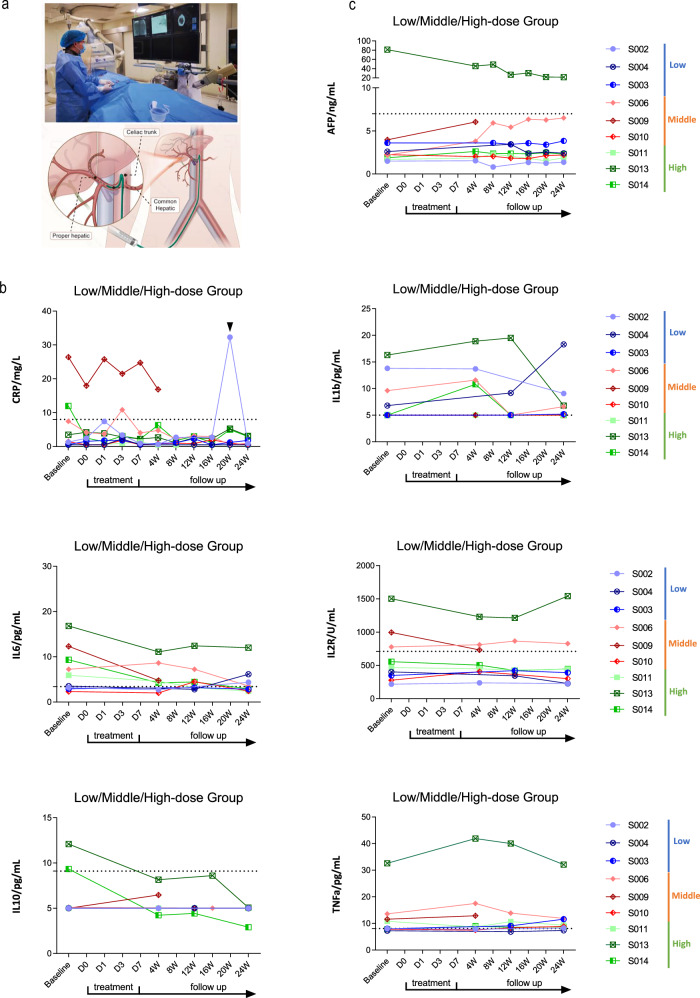
Safety and feasibility of HepLPC treatment in patients with cirrhosis. **a** Schematic diagram depicts the route of HepLPC transfusion. **b** The serum levels of CRP, IL-1β, IL-2R, IL-6, IL-10 and TNF-α in HepLPC-treated patients. Patient #2 presented with flu-like symptoms and displayed a transient high level of CRP. Dashed lines correspond to normal values. **c** Assessment of AFP before and after HepLPC treatment.

After establishing GMP-compliant manufacturing and demonstrating preclinical efficacy of HepLPCs in animal models, a single-arm, open-label clinical trial was initiated among liver cirrhosis patients to assess the treatment’s feasibility and safety. Between December 27, 2021, and December 14, 2022, 14 patients with varying etiologies of liver cirrhosis were recruited and screened. Nine patients received HepLPCs via trans-hepatic artery infusion in a dose-escalation manner: low (0.3–0.4 × 10⁶/kg), medium (1.5–2.0 × 10⁶/kg), and high (3.0–4.0 × 10⁶/kg) (Figs. 4, 5a). Due to the low immunogenicity and immunosuppressive effects observed in preclinical studies, no immunosuppressants were used post-treatment, and no immune rejections or inflammatory reactions were noted. Demographics and baseline characteristics are detailed in Supplementary Table S1. The nine patients had a mean age of 53 years (range 37–63) with a median Child-Pugh score of 7 (range 5–9). Etiologies included HBV-related cirrhosis (6 patients), primary biliary cholangitis (PBC) (2 patients), and HCV-related cirrhosis (1 patient), all under regular treatment. No patients experienced acute transfusion reactions or dose-limiting toxicity (DLT) during treatment.

Eight patients completed a 6-month follow-up. One patient in the medium-dose group with decompensated liver cirrhosis developed acute and severe cholecystitis and infection caused by gallstones, two months post-infusion. This led to acute-on-chronic liver failure (ACLF), necessitating liver transplantation 165 days after cell transfusion, causing the patient to withdraw prematurely from the trial (Fig. 4). The condition was considered unrelated to the treatment, given the transient presence of the infused cells. Notably, prior to acute cholecystitis, improvements in ammonia, activated partial thromboplastin time (APTT), Pre-ALB, TBIL and PLT levels were observed one month post-treatment, underscoring the safety of the infused cells. Consequently, this serious adverse event (SAE) was determined to be unrelated to the cell therapy. No other SAE occurred in other 8 out of 9 patients enrolled. A total of 61 adverse events (AEs), such as constipation, pruritus, nausea, and vomiting, were reported (Supplementary Table S2), with none attributed to HepLPCs or the infusion method, and all were well-tolerated. In terms of inflammation, serum markers including CRP, IL-1β, IL-2R, IL-6, IL-10, and TNF-α were monitored before and after cell transfusion (Fig. 5b). No patients developed cytokine release syndrome, as shown by CRP and IL-6 levels, consistent with safety analyses. Most patients demonstrated a decrease in IL-6 levels post-treatment, reinforcing the positive safety profile related to inflammation.

For hematologic toxicity evaluation, we assessed platelet levels, white blood cell (WBC) counts, and hemoglobin levels (Supplementary Fig. S7c). Typically, patients with cirrhosis and hypersplenism experience decreases in these parameters. However, after HepLPCs treatment, we did not observe such declines. Instead, hemoglobin levels significantly increased in almost all participants one month post-treatment and remained elevated throughout the study, with anemia completely reversed in patients #6 and #10. To assess potential liver malignancy, AFP levels and liver imaging (ultrasound and MRI) were monitored during follow-up. To date, no enrolled patient has demonstrated elevation in serum AFP level or developed liver neoplasms (Fig. 5c).

Taken together, our data strongly indicate that HepLPCs treatment, administered via trans-hepatic artery infusion without the use of immunosuppressants, is well tolerated in cirrhotic patients, presenting no additional risks and exhibiting promising safety outcomes.

### Liver function, coagulation, fibrosis indicators and images of patients after HepLPC treatment

**Fig. 6:**
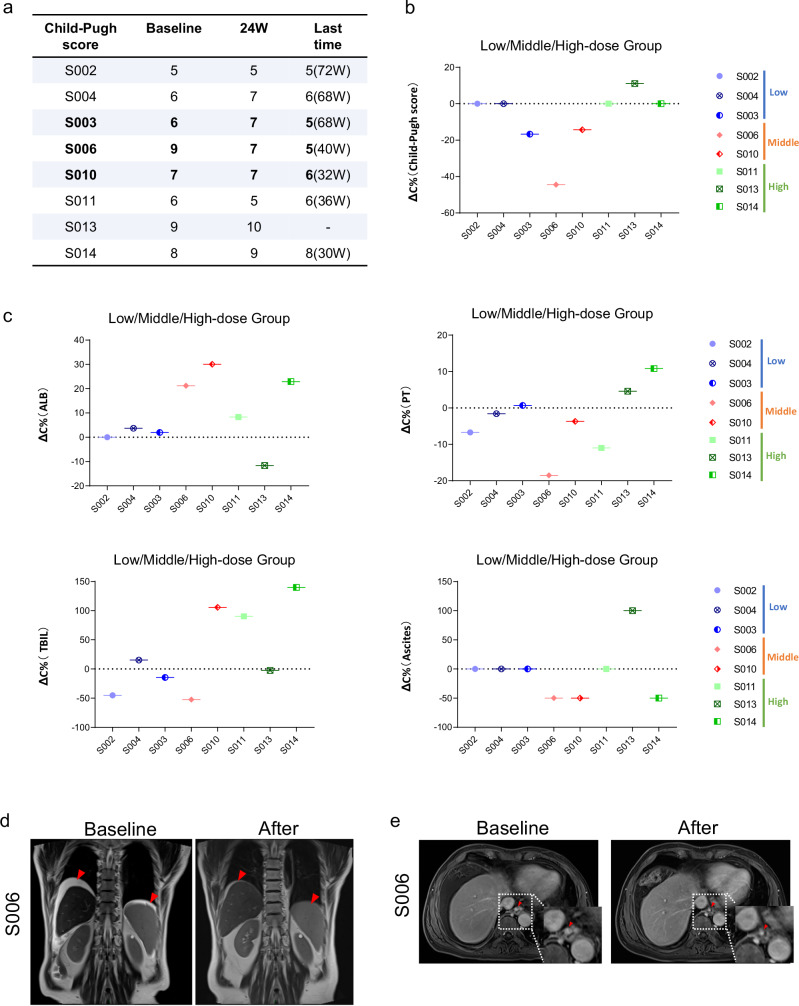
Efficacy of HepLPC treatment in patients with cirrhosis. **a** Assessment of Child-Pugh score before and after HepLPC treatment. **b,** **c** The changes in Child-Pugh score, ALB, PT, TB and degree of ascites in HepLPC-treated patients. ΔC = Value-Baseline, ΔC% = ΔC ÷ Baseline. **d** Ascites of patients #6 before and after HepLPCs treatment. **e** Diameter changes of the variceal vessels at the lower end of the esophagus in patient #6. Shown in the red box are indicators of improvement in cirrhosis.

Evaluation of clinical parameters after HepLPC treatment revealed that 3 out of 8 patients experienced decreased Model for End-Stage Liver Disease (MELD) and Child-Pugh scores (Fig. 6; Supplementary Fig. S8). A detailed analysis showed significant improvements in the medium-dose group, including reduced ascites, lower serum bilirubin levels, increased ALB levels, and improved prothrombin time (PT) (Supplementary Figs. S7, S9).

Markers of liver injury, such as ALT, AST, and TBIL remained stable post-treatment. Notably, patient #6, initially on the liver transplant waiting list, showed a significant decrease in TBIL, resulting in improved liver function and removal from the transplant list, with their Child-Pugh score dropping from 9 to 5. Furthermore, ALB and pre-ALB levels increased in most patients (6/8) (Fig. 6c; Supplementary Fig. S9), indicating enhanced liver synthesis function. Serum ammonia levels, used to assess detoxification ability, were consistently improved in all patients immediately post-treatment, persisting throughout follow-up.

Coagulation parameters, including PT, APTT, and fibrinogen, were evaluated. Both PT (5/8) and APTT (7/8) decreased, indicating recovery of coagulation function, while fibrinogen levels (5/8) increased significantly, demonstrating enhanced liver synthesis (Fig. S9). These improvements in coagulation parameters occurred four weeks post-treatment, contrasting with the rapid ammonia level improvement.

Liver stiffness measurements (LSMs) using elastography showed a gradual decrease in LSM values in four patients after HepLPC treatment, indicating improved liver stiffness (Supplementary Fig. S10a). Early serum markers for liver fibrosis — including hyaluronic acid (HA), type III procollagen (PC III), collagen IV (IV-C), and laminin (LN) — were also examined (Supplementary Fig. S10b). Patients with mild cirrhosis in the low-dose group exhibited a significant and consistent decrease in these markers, particularly HA levels, post-treatment. However, these markers did not consistently align with changes in advanced cirrhosis parameters in the medium and high-dose groups.

Additionally, two-dimensional shear wave elastography and MRI were conducted pre- and post-treatment. As shown in Supplementary Fig. S8b, 6 out of 8 patients experienced reduced spleen volume, suggesting an improvement in portal hypertension, consistent with previous clinical improvements. Notably, patient #6 showed enhanced liver function, improved coagulation, and hypersplenism resolution, with significant ascites reduction at 24 weeks (Fig. 6a–d). Additionally, the diameter of variceal vessels at the lower esophagus significantly decreased, indicating improved portal hypertension and reduced porto-systemic shunt (Fig. 6e).

To explore potential liver regeneration induced by HepLPC infusion, liver size changes were measured before and after treatment. Results showed that 6 out of 8 patients experienced increased liver volume post-treatment, suggesting hepatocyte proliferation in most cases (Supplementary Fig. S8b).

### The presence of HepLPCs in native liver

**Fig. 7:**
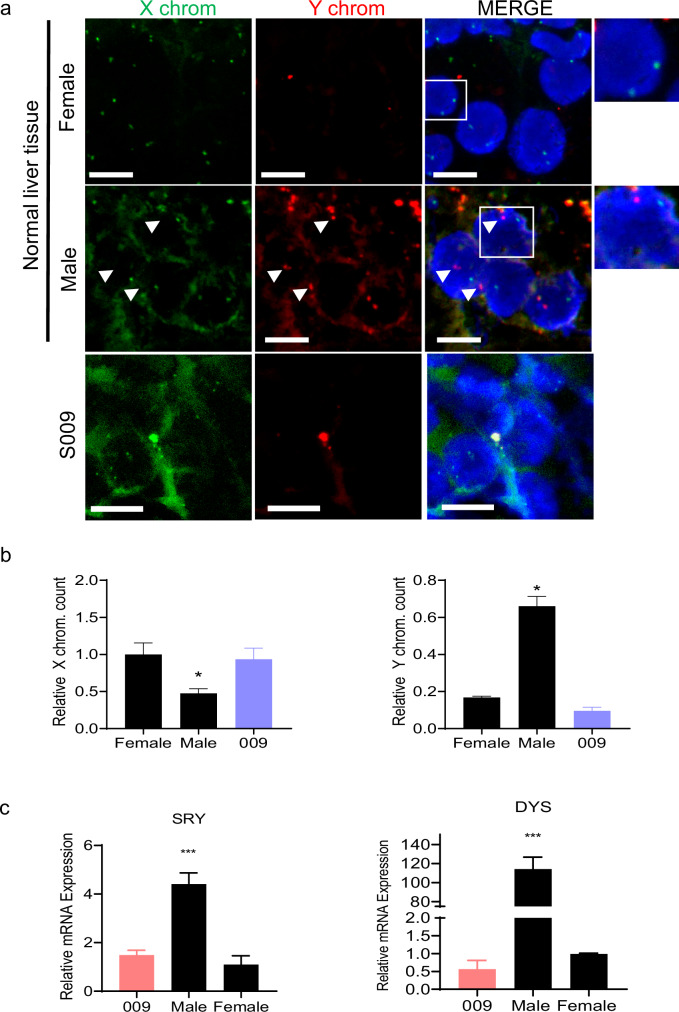
HepLPCs in liver tissues after transplantation. **a** X (green foci) and Y (red foci) chromosomes were detected with specific fluorescent-labeled probes. In the control group, two X chromosomes were detected in the female liver tissue, while X and Y chromosomes were detected in the male liver tissue. Scale bars: 20 μm. The arrows are pointing at the Y chromosome. **b** Quantification of FISH results for detecting both X and Y chromosomes. Relative counts of X and Y chromosomes were measured with Cellprofiler. Normal female liver tissue was regarded as a control group. *P < 0.05, Student’s *t*-test. **c** SRY and DYS14 levels were assessed using qPCR. Patient #9, a female recipient, was included in the analysis using their native liver tissue. Normal female liver tissue served as the negative control group, while male liver tissue served as the positive control group. ****P < 0.0001.

To investigate the presence of HepLPCs in native liver after transfusion, we examined their distribution in liver samples from participants who provided samples post-treatment (Fig. 7a). Patient #9, who withdrew from the clinical study and underwent liver transplantation, offered a valuable specimen. The liver tissue from patient #9 was subjected to fluorescent in situ hybridization (FISH) analysis to detect HepLPCs derived from male donors. Liver tissues from male and female donors served as positive and negative controls, respectively. The results showed no significant difference between the negative control and the liver from patient #9 (Fig. 7b), indicating no presence of HepLPCs in this participant’s native liver at the time of detection. Additionally, quantitative polymerase chain reaction (qPCR) analysis for donor-specific sequences (SRY and DYS14) confirmed that no meaningful Y chromosome-specific sequences were detected in the liver tissues of the two enrolled female recipients, compared with the female negative control and male positive control (Fig. 7c).

## Discussion

Liver is a vital organ that plays several essential roles, including detoxification, metabolism, digestion, and even producing blood clotting factors. However, when the liver is damaged, it can lead to a cascade of issues affecting various bodily functions^1^. Cirrhosis, regardless of its cause, can push patients to a stage where they might need a liver transplant, especially if they have developed complications or liver cancer. Unfortunately, there is a glaring mismatch between the number of potential donors and recipients. Approximately 21% of cirrhotic patients on the transplant waiting list either succumb to their condition or get removed due to worsening health^12^. To better the odds, managing the root cause of cirrhosis can significantly improve outcomes by slowing down its progression and possibly reversing some of the damage^13^. Alternative treatments to manage cirrhosis are therefore being developed, including cell transplantation^14,15^.

Cell transplantation holds great potential as an alternative treatment for liver fibrosis. Although the main goal of cell therapies is to alleviate liver fibrosis, they can also lead to additional beneficial effects such as reduced inflammation, enhanced angiogenesis, and increased liver regeneration^16,17^. Mesenchymal stem cells (MSCs) have taken center stage. Clinical trials in patients with liver cirrhosis have demonstrated the efficacy of these cells across multiple studies^18–21^, displaying notable antifibrotic effects. However, the effectiveness of MSC transplantation remains a topic of ongoing debate. A significant phase 2 trial in UK, involving multiple centers and using an open-label, randomized, controlled approach, concluded that stem cell transplantation did not show significant improvements in liver fibrosis or dysfunction. This contradictory evidence adds a layer of complexity to the ongoing discussion about the efficacy of this treatment approach^22^. On the other hand, fetal hepatocyte transplantation has emerged as another contender with promising results in numerous clinical studies for cirrhosis treatment^5^. However, ethical challenges and limitations in sourcing may pose hurdles to its widespread clinical use, prompting a need for more in-depth research into its mechanisms. Additionally, in patients with compensated cirrhosis (MELD 10–17), autologous monocyte-derived macrophages demonstrated a favorable safety profile and clinical promising trends, including a reduction in MELD scores at day 90, a lower incidence of liver-related SAEs, and favorable anti-inflammatory cytokine changes, though the primary endpoint narrowly missed statistical significance (ΔΔMELD –0.87, *p* = 0.06). The findings of macrophage therapy underscore the emerging feasibility of cell-based cirrhosis interventions, while our HepLPC findings advance this field by demonstrating another safety-supported, biologically plausible regenerative option^23^. In this study, we developed a hepatocyte-derived product using HepLPCs manufactured under GMP conditions. These cells demonstrated a close clustering with fetal hepatocytes in their gene expression profiles. To investigate the paracrine effects of HepLPCs, we co-cultured them with LX2 cells or primary hepatocytes. In vitro experiments showed that HepLPCs’ supernatant reduced Col1a1 expression in LX2 cells and promoted primary hepatocyte proliferation. These effects were blocked by an MMP inhibitor and HGF/c-MET antagonists, suggesting that MMPs and HGF may play crucial roles in mitigating fibrotic responses and promoting liver regeneration. Before being applied to patients, HepLPCs showed remarkable effectiveness in animal models of liver cirrhosis, as evidenced by significant mitigation of fibrosis as well as marked improvement in liver function and regeneration. The subsequent outcomes from this first-in-human study involving nine patients underscored the safety and feasibility of HepLPC treatment in patients with cirrhosis, opening up vast possibilities for clinical application.

Herein, the administered dose of HepLPCs (0.3–4.0 × 10^6^/kg) was similar to that applied in previous studies involving hepatic stem cells^24^ and MSC treatments for metabolic liver diseases^25,26^. The safety profile from our clinical study demonstrates that HepLPCs are safe and non-immunotoxic for human use. Notably, no treatment-related AEs or signs of immunotoxicity were observed in any of the patients, which is particularly remarkable given the absence of immunosuppressive therapy. Across the dosage spectrum, there were no treatment-related AEs or SAEs, with other AEs aligning with expectations in patients with liver cirrhosis. Remarkably, participants exhibited no rise in transaminase levels or inflammation cytokines after HepLPC treatment, highlighting the absence of HepLPC-induced hepatic injury or immunotoxicity. This safety profile aligns with our pre-clinical findings regarding the inherent low immunogenicity of HepLPCs. For example, HepLPCs are characterized by an absence of expression of MHC class II antigens, including HLA-DP, DQ, and DR. They also have a suppressive effect on T cell proliferation, particularly the T cell subsets Th1 and Th17, and downregulate their expression of the pro-inflammatory cytokine TNF-α. Importantly, under inflammatory treatment, the expression of HLA-DR/DP/DQ did not show any significant increase. Collectively, these findings suggest that HepLPCs can be safely applied without the need for additional immunosuppressants.

Another critical aspect to consider regarding the safety of this clinical study is the administration route of HepLPCs. Various cell delivery methods have been documented in the context of liver diseases, among which splenic transplantation and hepatic portal vein route are typically used. However, the procedures are associated with risks of portal vein hypertension, bleeding, or thrombosis^27^. Alternative routes, such as the intra-hepatic artery as demonstrated in this study, offer advantages including higher blood flow velocity, reduced thrombosis formation, and lower portal hypertension, along with enhanced implantation efficiency and persistence compared to the portal vein route. Up to date, no bleeding or clotting events have been found after HepLPCs treatment, suggesting its feasibility and safety in future clinical applications.

Although there was no significant improvement in the Child-Pugh and MELD scores of the enrolled patients, and no histological confirmation of cirrhosis changes from liver biopsies, some patients showed improvements in liver biochemistry, coagulation, and portal hypertension parameters following HepLPC treatment. Noteworthy improvements were observed in synthetic liver function markers, including pre-ALB, APTT, and fibrinogen, after HepLPC treatment. Detoxification analysis further revealed reductions in bilirubin and ammonia levels in the majority of patients. HepLPC treatment significantly improved portal hypertension manifestations, including splenomegaly reduction, ascites resolution, portosystemic collateral regression, and liver regeneration, supporting its disease-modifying role in cirrhosis. Specifically, significant improvements in synthetic liver parameters began as early as 4 weeks after HepLPC treatment, and intriguingly, no donor cells were detected in the native liver months after treatment, indirectly highlighting the paracrine effects of HepLPCs on liver regeneration.

For instance, patient #6, initially on the liver transplant waiting list due to decompensated cirrhosis caused by HBV, was eventually removed from the list as his Child-Pugh score decreased from 9 to 5 at week 40. The substantial improvements in liver function, coagulation, and hematology were accompanied by consistent amelioration of ascites and porto-systemic shunt. In contrast, patient #9 with PBC-induced cirrhosis developed acute and severe cholecystitis and infection from gallstones two months post-treatment, and experienced ACLF, ultimately leading to liver transplantation 165 days after HepLPC treatment. Notably, before this cholecystitis-induced SAE, serum total bilirubin and ammonia had visibly decreased, along with platelet restoration within one month (Supplementary Fig. S11). These results suggest that the treatment had beneficial effects on patient #9 before the onset of the SAE. However, infection markers, such as CRP, began to sharply rise in the second month, indicating an underlying infection occurred that may have contributed to the subsequent development of ACLF. Consequently, this SAE was determined to be unrelated to the cell therapy. Patient #11, also with PBC-induced cirrhosis but without cholestasis, showcased comprehensive improvements in liver function and coagulation results without any SAE during follow-up. Importantly, the absence of SAEs in 8 out of 9 patients confirmed the safety of the trial. These findings also support the potential effectiveness of HepLPC treatment, emphasizing the need for careful scrutiny of inclusion criteria regarding the primary cause of cirrhosis and the disease state in future clinical studies, with consideration of liver histology.

Nevertheless, further mechanistic studies are imperative to elucidate how HepLPCs promote tissue regeneration while avoiding pro-fibrotic/inflammatory cascades. Furthermore, variability in responses to HepLPC treatment was evident across patients, even among those with the same dose range or etiology. Randomized controlled trials will be carried out in the near future to systematically assess the impact of various variables, including etiology, clinical management, and cirrhosis severity.

In summary, our study demonstrates that trans-hepatic artery infusion of HepLPCs in cirrhosis patients is safe, feasible, and potentially effective in managing liver cirrhosis. This novel therapeutic approach holds significant promise for clinical practice in treating liver cirrhosis. The encouraging outcomes provide a strong rationale for advancing to the next phase of clinical trials, extending the potential application beyond cirrhosis to acute-on-chronic liver failure.

Several limitations existed in this clinical study. First, percutaneous liver biopsy before and after HepLPC treatment was not mandatorily conducted because of the high risk of this procedure on decompensated liver cirrhosis. Second, we did not directly evaluate the levels of portal hypertension. In our further clinical trial, a transjugular liver biopsy and hepatic venous pressure gradient will be performed at the same time to show the severity of cirrhosis and hypertension with more solid evidence. Furthermore, indocyanine green retention at 15 min (ICG-R15), as a non-invasive marker of the functional hepatic reserve, appears to be correlated with the severity of portal hypertension and cirrhosis^28^, which will also be included in our assessment parameters in the following clinical studies.

## Materials and methods

### Cell production

Clinical manufacturing of HepLPCs was performed in the GMP facility of Cryowise Technology, China. First, hepatocytes were isolated from non-transplantable liver tissues using a customized two-step collagenase perfusion technique. The donor tissues, sourced through approved channels with written informed consent provided by donors or their legal representatives, ensured the integrity of the process. The viability of these hepatocytes was over 70% as determined by Trypan blue staining (Sigma-Aldrich). The cells were plated on CellBIND^®^ surface 150 cm^2^ cell culture flasks (Corning) at 0.1–0.2 × 10^5^ cells/cm^2^ within an optimized culture condition containing animal-free growth factors based on TEM as described previously^7^. Briefly, TEM composed of DMEM/F12 supplemented with human platelet lysate (HPL) and the following growth factors or small molecules: 20 ng/mL HGF, 20 ng/mL EGF, 10 μM Y27632, 3 μM CHIR99021, 1 μM A8301, 1 μM S1P and 5 μM LPA. Over a 14-day period, under the induction of small molecules cocktail, hepatocytes were progressively converted into an expandable liver progenitor cell state (designated as HepLPCs). These HepLPCs were dissociated to single cells using TrypLE Express (Invitrogen), followed by continuous culture on CellBIND^®^ surface 150 cm^2^ flasks. HepLPCs consistently exhibited the key features of liver progenitor cells with an appropriate PDL exceeding 2. Through successive rounds of expansion using hyper-flasks (Corning), an impressive yield of approximately 0.5–1×10^10^ HepLPCs was achieved. These cells were then harvested, resuspended in a compound electrolyte solution supplemented with 5% DMSO and HSA, and packaged in cryopreservation bags (Miltenyi Biotec). Finally, the cryopreservation bags were placed in a Thermo Scientific Forma CryoMed Controlled Rate Freezer, and subsequently transferred to a liquid nitrogen tank and managed in strict accordance with GMP standards.

### Study design

This investigator-initiated clinical study, generously funded by Shanghai Cryowise Technology Co., Ltd., marks a significant advancement in addressing the unmet medical needs of liver cirrhosis patients. The study for both donors and recipients received ethical approval from the Ethics Committee of Renji Hospital, Shanghai Jiao Tong University School of Medicine (KY2020-084), and has been meticulously registered on both ClinicalTrials.gov (NCT04806581) and the Chinese Clinical Trial Registry (ChiCTR2000032383).

This first-in-human trial was conducted using a standard 3 + 3 dose escalation design at Renji Hospital, Shanghai Jiao Tong University School of Medicine, Shanghai, China. The primary endpoint included safety, which was assessed based on the incidence of adverse events and laboratory tests, including hematology and blood chemistry. The secondary endpoint was preliminary efficacy including the recovery of liver function, coagulation, and hematology parameters, as well as structural improvement of cirrhosis through imaging evaluations.

All participants were recruited from the outpatient service of Renji Hospital between December 27th, 2021 and December 14th, 2022. Nine adult participants with liver cirrhosis of diverse etiologies and a Child-Pugh score within 9 were enrolled after obtaining informed written consent. Inclusion criteria were as follows: (1) age 18–70 years; (2) liver disease etiology including alcohol-related liver disease, auto-immune liver diseases, non-alcoholic fatty liver disease, or treated chronic hepatitis B or C (sustained viral response); (3) liver cirrhosis (diagnosed by liver biopsy, or clinical and imaging evidence consistent with cirrhosis); (4) anatomically suitable for percutaneous transhepatic proper artery puncture. Exclusion criteria were as follows: (1) history of severe cirrhosis complication within the preceding 4 weeks (portal hypertensive bleeding, spontaneous bacterial peritonitis, or refractory ascites); (2) liver or extrahepatic malignancy; (3) pregnancy or breastfeeding; (4) concurrent acute extrahepatic illnesses that may compromise patient safety during the trial. The HepLPCs were produced in a GMP-accredited facility. Each group of three participants received a single infusion of cells, with doses stratified as low, medium, or high, delivered via a proper hepatic artery approach.

### Study assessments

Throughout the infusion process, participants were monitored closely and observed overnight (within 24 h), all within the confines of the Clinical Research Facility. In anticipation of severe reactions, special arrangements were made with the intensive care unit. On days 0, 1, 3, and 7, as well as at subsequent intervals of 4, 8, 12, 16, 20, and 24 weeks following HepLPCs infusion, a comprehensive set of assessments was conducted.

To assess potential toxicity, a battery of tests, including full blood count, renal function, electrolytes, liver function tests, lipid profile, and coagulation series, was performed on days 0, 1, 3, and 7. During the initial one-month follow-up visits after cell infusion, safety and DLT were assessed. DLT criteria were established based on accepted measures^29–31^: serum creatinine, ≥ 1.5-fold from baseline; hemoglobin, 1.5-fold ≤ baseline; platelets, 3-fold from baseline; total bilirubin, > 3-fold from baseline; MELD score, > 4 points from baseline.

Thereafter, participants underwent scheduled follow-up evaluations at weeks 4, 8, 12, 16, 20, and 24 after HepLPCs infusion, including routine and biomarker blood tests, abdominal ultrasound, transient elastography, Gd-EOB-DTPA-enhanced MRI of the liver and Performance Score assessment (Supplementary Table S3). Various serological biomarker tests, such as HA, PC III, IV-C, and LN, were employed for assessing liver fibrosis. Liver function was evaluated using Child-Pugh scores, the established clinical scores derived from objective variables (e.g., serum bilirubin, ALB and INR), along with the grade of hepatic encephalopathy and ascites. Serum cytokines, including IL-1β, IL-2R, IL-6, IL-10 and TNF-α were analyzed to evaluate immune responses. Importantly, even 24 weeks after HepLPCs treatment, continuous monitoring through clinic follow-ups persisted for a span of 2 years. More details of materials and methods are provided in the Supplementary information.

## Supplementary information

SUPPLEMENTAL MATERIAL
