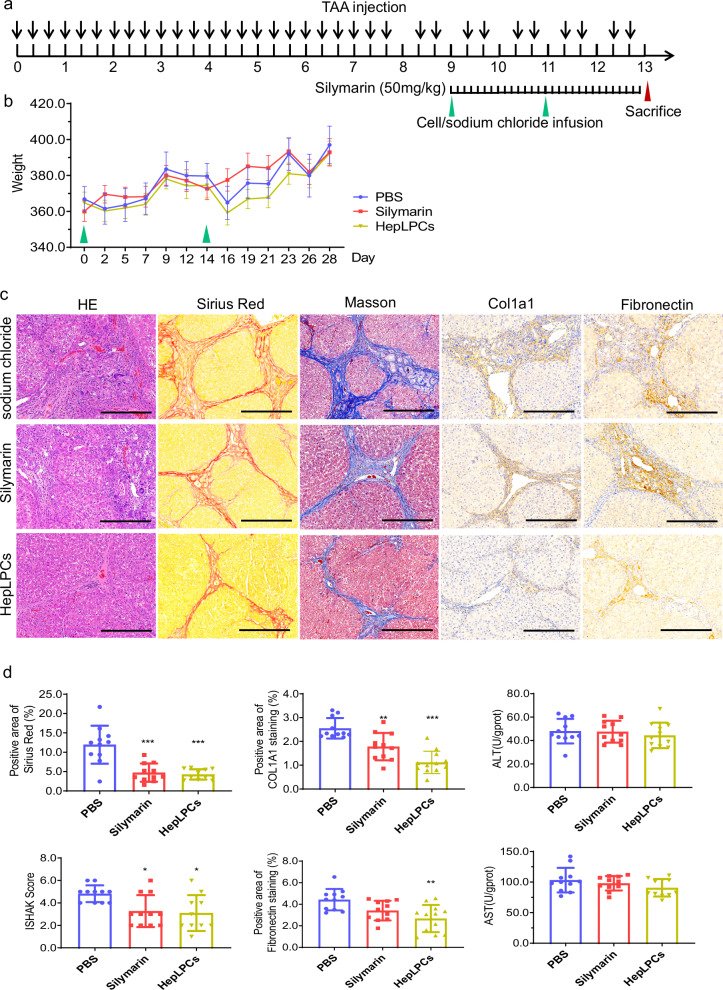# Author Correction: Treatment of liver cirrhosis using hepatocyte-derived liver progenitor-like cells: a prospective, open-label, single-arm, safety trial

**DOI:** 10.1038/s41421-025-00862-5

**Published:** 2025-12-10

**Authors:** Kang He, Xue-Jing Zhu, Yao-Ping Shi, Wei-Jian Huang, Tai-Hua Yang, Zhi-Feng Xi, Qi-Gen Li, Han-Yong Sun, Li-Jun Qian, Xiao-Song Chen, Pei-Ying Li, Xu Zhou, Gui-Ying Gu, Fan Li, Wen-Ming Liu, Cai-Yang Chen, Jie Zhao, Hong-Ping Wu, Fang-Rong Yan, Michael Ott, Amar Deep Sharma, Hui Liu, Wei-Feng Yu, Bo Zhai, He-Xin Yan, Qiang Xia

**Affiliations:** 1 Department of Liver Surgery, Renji Hospital, School of Medicine, Shanghai Jiao Tong University, Shanghai, China; 2 Celliver Biotechnology Inc., Shanghai, China; 3 Department of Interventional Oncology, Renji Hospital, School of Medicine, Jiao Tong University, Shanghai, China; 4 Department of Anesthesiology and Critical Care Medicine, Renji Hospital, School of Medicine, Shanghai Jiao Tong University, Shanghai, China; 5 Key Laboratory of Anesthesiology (Shanghai Jiao Tong University), Ministry of Education, Shanghai, China; 6 Department of Radiology, Renji Hospital, School of Medicine, Shanghai Jiao Tong University, Shanghai, China; 7 Department of Infectious Diseases, Renji Hospital, School of Medicine, Shanghai Jiao Tong University, Shanghai, China; 8 Department of Clinical Laboratory, Eastern Hepatobiliary Surgery Hospital, Naval Medical University, Shanghai, China; 9 Department of Biostatistics, China Pharmaceutical University, Nanjing, Jiangsu China; 10 Department of Gastroenterology, Hepatology and Endocrinology, Hannover Medical School, Hannover, Germany; 11 Department of Hepatic Surgery, Eastern Hepatobiliary Surgery Hospital, Naval Medical University, Shanghai, China

**Keywords:** Regeneration, Reprogramming

Correction to: *Cell Discovery* 10.1038/s41421-025-00831-y published online 05 November 2025

In the originally published version of this article, Fig. 2d (“Positive area of COL1A1 staining”) contained a bar chart exported from an earlier analysis draft. This panel has been replaced with the chart generated from the final, pre-specified analysis pipeline. This correction does not affect the statistical results, the textual interpretation, or the conclusions of the study.

**Incorrect Figure 2** 
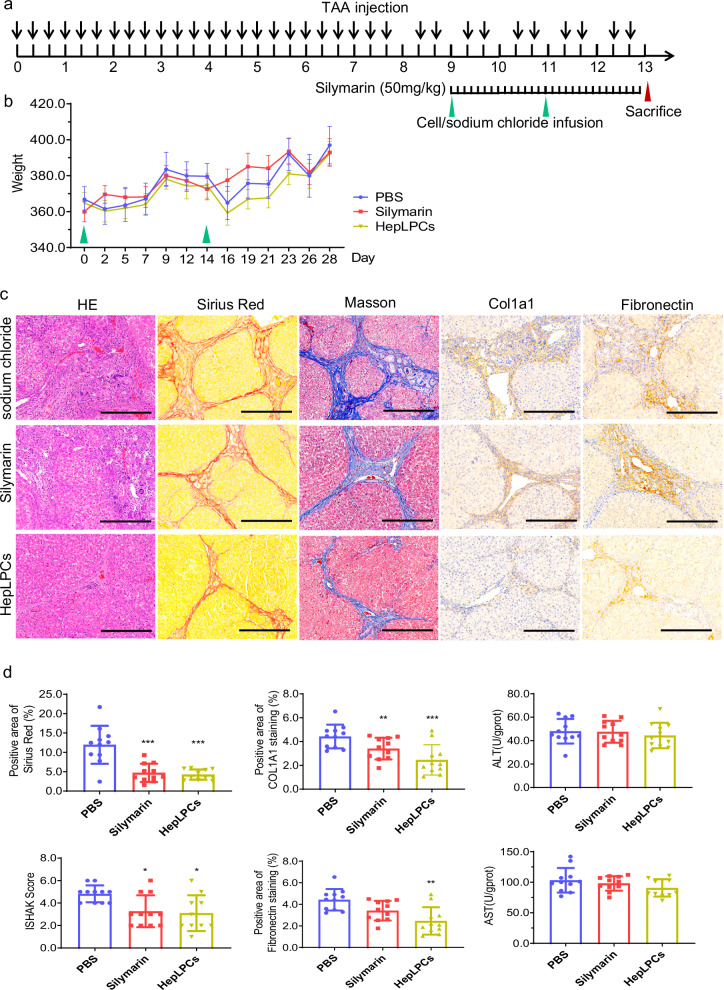


**Correct Figure 2**