# Supplementary material for: Treatment of liver cirrhosis using hepatocyte-derived liver progenitor-like cells: a prospective, open-label, single-arm, safety trial
Source: Cell Discov. 2025 Nov 5;11:88. doi: 10.1038/s41421-025-00831-y (PMC12589444; doi:10.1038/s41421-025-00831-y)
Supplement: Supplementary file 1 — SUPPLEMENTAL MATERIAL [file 41421_2025_831_MOESM1_ESM.docx]

Treatment of liver cirrhosis using hepatocyte-derived liver progenitor-like cells (HepLPCs): a prospective, open label, single arm, safety trial

Kang He^1*^, Xue-Jing Zhu^2*^, Yao-Ping Shi^3*^, Wei-Jian Huang^2,4,5*^, Tai-Hua Yang^1*^, Zhi-Feng Xi^1^, Qi-Gen Li^1^, Han-Yong Sun^1^, Li-Jun Qian^6^, Xiao-Song Chen^7^, Pei-Ying Li^4,5^, Xu Zhou ^4,5^, Jia-Xin Li^1^, Ren-Jie Huang^2^, Wen-Ming Liu^4,5^, Cai-Yang Chen^4,5^, Jie Zhao^1^, Hong-Ping Wu^8^, Fang-Rong Yan^9^, Michael Ott^10^, Amar Deep Sharma^10^, Wei-Feng Yu^4,5#^, Bo Zhai^3#^, He-Xin Yan ^2,4,5#^ and Qiang Xia^1#^

**Affiliations:**

^1^Department of Liver Surgery, Renji Hospital, Shanghai Jiao Tong University School of Medicine, China;

^2^Celliver Biotechnology Inc., Shanghai, China;

^3^Department of Interventional Oncology, Renji Hospital, Shanghai Jiao Tong University School of Medicine, China;

^4^Department of Anesthesiology and Critical Care Medicine, Renji Hospital, Shanghai Jiao Tong University School of Medicine, China;

^5^Key Laboratory of Anesthesiology (Shanghai Jiao Tong University), Ministry of Education, China;

^6^Department of Radiology, Renji Hospital, Shanghai Jiao Tong University School of Medicine, China;

^7^Department of Infectious Diseases, Renji Hospital, Shanghai Jiao Tong University School of Medicine, China;

^8^International Cooperation Laboratory on Signal Transduction, Eastern Hepatobiliary Surgery Hospital, Second Military Medical University, Shanghai, China;

^9^Department of Biostatistics, China Pharmaceutical University, Nanjing, China;

^10^Department of Gastroenterology, Hepatology and Endocrinology, Hannover Medical School, Hannover, Germany.

^*^ These authors contributed equally to this work.

^#^ Corresponding authors: Wei-Feng Yu, Email: ywf808@yeah.net

Bo Zhai, Email: zhaiboshi@sina.com

He-Xin Yan, Email: hexinyw@163.com

Qiang Xia, Email: [xiaqiang@shsmu.edu.cn](mailto:xiaqiang@shsmu.edu.cn)

**Supplementary Materials & Methods**

**Key Materials Table**

| REAGENT or RESOURCE | SOURCE | IDENTIFIER |
| --- | --- | --- |
| Antibodies | | |
| Anti-HNF-4-alpha | Abcam | ab41898 |
| Human Serum Albumin PE-conjugated Antibody | R&D | IC1455P |
| IgG H&L (DyLight® 488) | Abcam | ab96879 |
| PE Mouse Anti-Human CD44 | BD | 550989 |
| PE-Cy^TM^7 Mouse Anti-Human CD90 | BD | 561558 |
| PE anti-human CD155 (PVR) | Biolegend | 337610 |
| APC anti-human HLA-DR, DP, DQ | BioLegend | 361714 |
| Alexa Fluor®700 Mouse anti-Human CD34 | BD | 561440 |
| FITC Mouse Anti-Human CD45 | BD | 555482 |
| Alexa Fluor® 647 anti-human INF-a | BioLegend | 502916 |
| Anti-Collagen I | Abcam | ab270993 |
| PE anti-human CD8a Antibody | Biolegend | 300908 |
| PE-Cy7 Mouse Anti-Human IFN-y(B27) | BD | 557643 |
| PHA | DaHui bio | S0010 |
| CFSE | Thermo | C34570 |
| APC anti-human IL-17A | Biolegend | 512334 |
| Alexa Fluor® 647 Mouse anti-Human FoxP3 | BD | 560045 |
| Leukocyte Activation Cocktail, with BD GolgiPlugTM | BD | 550583 |
| Fixation/Permeabilization Solution Kit | BD | 554714 |
| Chemicals, peptides, and recombinant proteins | | |
| DMEM/F12 | Gibco | 11320033 |
| Stemulate® human platelet lysate (HPL) | exton Biotechnologies | PL-SP-500 |
| Recombinant Human HGF | Pepro Tech | 100-39 |
| Recombinant human EGF | Pepro Tech | 100-15R |
| Y27632 | TargetMol | T1870 |
| CHIR99021 | TargetMol | T2310 |
| A8301 | TargetMol | T3031 |
| LPA | ENZO | BML-LP100 |
| Critical commercial assays | | |
| Human HGF ELISA Kit | Multi Sciences | EK1H01-96 |
| Human MMP-1 (Matrix Metalloproteinase 1) ELISA Kit | Sangon Biotech | D711193-0096 |
| Human MMP-3 (Matrix Metalloproteinase 3) ELISA Kit | Sangon Biotech | D711195-0096 |
| Deposited data | | |
| HepLPCs and PHH RNA-seq data | This paper | GEO: GSE248227 |
| Software and algorithms | | |
| GraphPad Prism8 | GraphPad Software | Version 8.0.2 |
| ImageJ | NIH | https://imagej.nih.gov/ij/ |
| FlowJo software | Tomy Digital Biology | FlowJo 7.6 |

**Lead contact**

Further information and requests for resources and reagents should be directed to and will be fulfilled by the lead contact, xiaqiang@shsmu.edu.cn

**Materials availability**

All stable reagents generated in this study are available from the lead contact without restriction.

**Co-culture Experiment:**

For the lymphocyte proliferation inhibition assay, HepLPCs are seeded onto cell culture plates and incubated under appropriate conditions for approximately 24 hours. The next day, PBMCs are labeled with CFSE, and after the addition of PHA to promote mitosis and IL-2 activation, PBMCs and HepLPCs are co-cultured for 3-4 days. Activated lymphocytes labeled with CFSE will proliferate in response to stimulation, and the changes in CFSE signal intensity are detected by flow cytometry to assess the inhibitory effect of HepLPCs on lymphocyte proliferation. For the TNFα secretion assay, PBMCs labeled with Alexa Fluor® 647 anti-human TNF-α are analyzed by flow cytometry to detect changes in TNF-α signal, thus determining the effect of HepLPCs on the secretion of TNF-α by PBMCs. For Th1 and Th17 cell detection, PBMCs are stained with surface markers CD3 and CD8, as well as intracellular markers IFN-γ and IL-17A, followed by flow cytometric analysis to measure changes in cell proportions. For Treg cell detection, PBMCs are stained with surface markers CD3, CD4, and CD25, along with intracellular marker FoxP3, and analyzed by flow cytometry to assess the changes in cell proportions.

For the co-culture with LX-2 and hepatocytes, the medium used to culture HepLPCs is replaced with DMEM high-glucose serum-free medium, and further incubated for 72 hours. The conditioned media was collected under these conditions will be used for the indirect co-culture with LX-2 cells or hepatocytes. Following treated with 2.5 ng/mL TGF-β1 for 24 hours, LX-2 cells is replaced with the conditioned media in the experimental group, while the control group is replaced with DMEM high-glucose serum-free medium. After immunofluorescent staining for Collagen I, imaging and analysis are performed using the high-content imaging system (Molecular Devices, Image X Press Micro confocal system).To further verify that MMP1 is the effective component of the HCL001 cell injection, this experiment uses the MMPs inhibitor Marimastat (BB-2516) added simultaneously to the LX-2 cells at concentrations of 20 μM, which will be utilized for the indirect co-culture with LX-2 cells. For hepatocytes, the control group and experimental group are changed to their respective culture media, while MK2461 (HGF/c-MET signaling antagonists, 10 μM) added to the hepatocytes simultaneously, and incubated for an additional 48 hours. After this period, microscopic images are taken, followed by digestion and centrifugation for cell counting.

Twenty-four hours after plating, HepLPCs were incubated for an additional 24 hours with or without a cocktail of pro-inflammatory cytokines, specifically 20 ng/mL IL-1β, 10 ng/mL IFN-γ, and 50 ng/mL TNF-α. After this treatment, the cells were washed twice with sufficient volumes of PBS to remove the cytokines and then incubated for another 24 hours in DMEM/F12. Flow cytometry analysis revealed that HepLPCs did not express HLA-DR/DP/DQ, regardless of inflammatory treatment.

**RNA sequencing and bioinformatics analysis**

Total RNA was isolated using the RNeasy mini kit (Qiagen, Germany), quantified by NanoDrop ND-2000 spectrophotometer (Thermo Fisher Scientific, Waltham, MA, USA) and integrity was determined by the Agilent 2100 system and RNA 6000 Nano kit (Agilent Technologies, Santa Clara, CA, USA). RNA-seq was conducted by Shanghai NewCore Biotech. Co., Ltd. Briefly, paired-end libraries were constructed using TruSeq Stranded mRNA LTSample Prep Kit (Illumina, San Diego, CA, USA), according to the manufacturer's instructions. Libraries were sequenced on an Illumina platform (Novaseq 6000, Illumina), and 151 bp paired-end reads were generated. Original data were uploaded to the Gene Expression Omnibus database. Bioinformatics data analysis is provided by Shanghai NewCore BioTech Co., Ltd. The fastp software (v0.20.0) is used to trim adapters and remove low-quality reads, obtaining high-quality clean reads. The STAR software (v2.7.9a) is used to align the high-quality clean reads to the human reference genome (hg38). The featureCounts software (v2.0.2) is used to obtain mRNA gene raw read counts as mRNA expression values. The DESeq2 software (v1.30.1) is used for normalization and calculation of fold changes and p-values to filter differentially expressed mRNAs. The mRNA is annotated using the GTF annotation database (Ensembl v104). The clusterProfiler R package (v3.18.1) is employed for Gene Ontology and KEGG Pathway enrichment analysis of differentially expressed mRNAs. The rMATS software (v4.1.1) is used to predict differential alternative splicing events between two groups of samples.

**Karyotyping and Genetic Stability Analysis**

DNA libraries for WGS analysis were constructed from 200 ng of genomic DNA using VAHTS Universal Pro DNA Library Prep Kit for Illumina. The libraries were sequenced with pair-end (2 × 150 bp) runs using Illumina novaseg sequencers based on 100× base coverage depth. Using Control-FREEC software, we calculated the CNV in the HepLPCs sample relative to the PHH sample. This genome-wide sequencing analysis allowed us to examine the log2DNA copy ratio across the genome, represented by yellow dots. We established clear thresholds for identifying DNA amplifications and deletions based on the DNA copy ratio: amplifications were defined as a DNA copy ratio greater than 1.5, and deletions as a ratio less than 0.5.

**Animal study design**

For TAA-induced cirrhosis models, rats were intraperitoneally administered with Thioacetamide (TAA, 200 mg/kg) three times per week for 13 weeks. After 8 weeks, the rats were divided into three groups. In the HepLPCs groups, the rats underwent portal vein transplantation of HepLPCs every 2 weeks (two times in total) 3×10^6^ cells/kg per rat (n = 12). In the control group, negative control rats received 3 mL/kg of 0.9% sodium chloride (n = 12), while positive control rats received daily treatment with 50 mg/kg Silymarin for 4 weeks (n = 12). After 10 weeks, all rats were sacrificed, and their blood samples and liver tissues were collected for biochemical and histological analyses. All animal procedures were performed in accordance with the Reporting of In Vivo Experiments (ARRIVE) guidelines for the care and use of laboratory animals, and were approved by the Institutional Animal Care and Use Committee of Shanghai Model Organisms Center, Inc. (IACUC2019-0027-06).

**Repeated-dose toxicity test of HepLPCs in crab-eating macaques:**

In this study, 36 crab-eating macaques were randomly divided into a solvent control group and low- and high-dose HepLPC injection groups (doses of 0, 1×10^6^, and 4×10^6^ cells/kg, respectively). Each group consisted of 10, 12, and 14 animals, respectively, with an equal number of males and females. The animals in each group were given 0.4 mL/kg of vehicle control (cell cryopreserved solution) or corresponding concentration of HepLPC injection by hepatic artery perfusion once every 4 weeks, and the drug was given twice in total for 4 weeks). After the treatment period, observation continued for 22 weeks. The animals were dosed in batches, with the first day of dosing in each batch defined as Day 1 (D1), with dosing occurring on D1 and D29.

**Quantitative real-time PCR (qPCR)**

The total RNA of human and rat tissues was extracted using TRIzol reagent (Invitrogen) according to the manufacturer’s protocols. Real-time PCR analyses were performed using the ABI PRISM 7500 sequence detection system (Applied Biosystems) and SYBR Green PCR kit (Roche). Gene transcription was evaluated using the ΔΔCt method normalized to the housekeeping gene actin beta (ACTB). Primer sequences are listed as follows:

SRY forward: 5'-TCC TCA AAA GAA ACC GTGCAT-3'

SRY reverse: 5'-AGA TTA ATG GTT GCT AAG GACTGG AT-3'

DYS14 forward: 5'-GGG CCA ATG TTG TAT CCT TCTC-3’

DYS14 reverse: 5'-GCC CAT CGG TCA CTT ACA CTTC-3'）.

**Fluorescent in situ hybridization (FISH)**

DNA FISH experiments for detecting the X and Y chromosomes were performed using a commercially available CEN X/Y dual color probe and related kit in ZytoVision (https://www.zytovision.com/), according to the manufacturer’s protocol. Briefly, liver samples were collected as previously described. Paraffin-embedded liver tissue slides were prepared at Renji Hospital. After dewaxing with Xylene and hydration with ethanol, antigen retrieval was performed and endogenous peroxidase was removed with Pepsin Solution. After washing and dehydration with graded ethanol, probe incubation was performed at 72 °C for 5 min and hybridized overnight at 37 °C. Then, the slides were washed and dehydrated again, followed by counterstaining with DAPI. High-resolution images were acquired using a ZEISS confocal microscope, and data analysis was performed with Cellprofiler.

**Statistics**

In the study focused on safety and feasibility outcomes, a descriptive analysis was conducted. For enhanced clarity, secondary outcomes were thoughtfully illustrated through graphical representations, both by dose and as changes from baseline. Numerical data were presented as mean±standard deviation (SD), providing a concise yet informative snapshot of the results. We adopted the Student’s t distribution to conduct hypothesis test assuming a normal distribution of the data. Ensuring a proactive approach to participant safety, a dedicated safety report was initiated promptly, specifically focusing on the results obtained at week 4 for the initial participants. Our commitment to data integrity involved a rigorous electronic database lock following thorough quality checks. Subsequent analyses were conducted with precision, adhering to the highest standards of scientific inquiry. Importantly, our commitment to transparency and participant welfare was evident in the reporting of all adverse events encountered during the study. A thorough 100% quality check of the collected data was executed, leaving no room for missing or ambiguous information.

**Supplementary Figures & Tables**

**
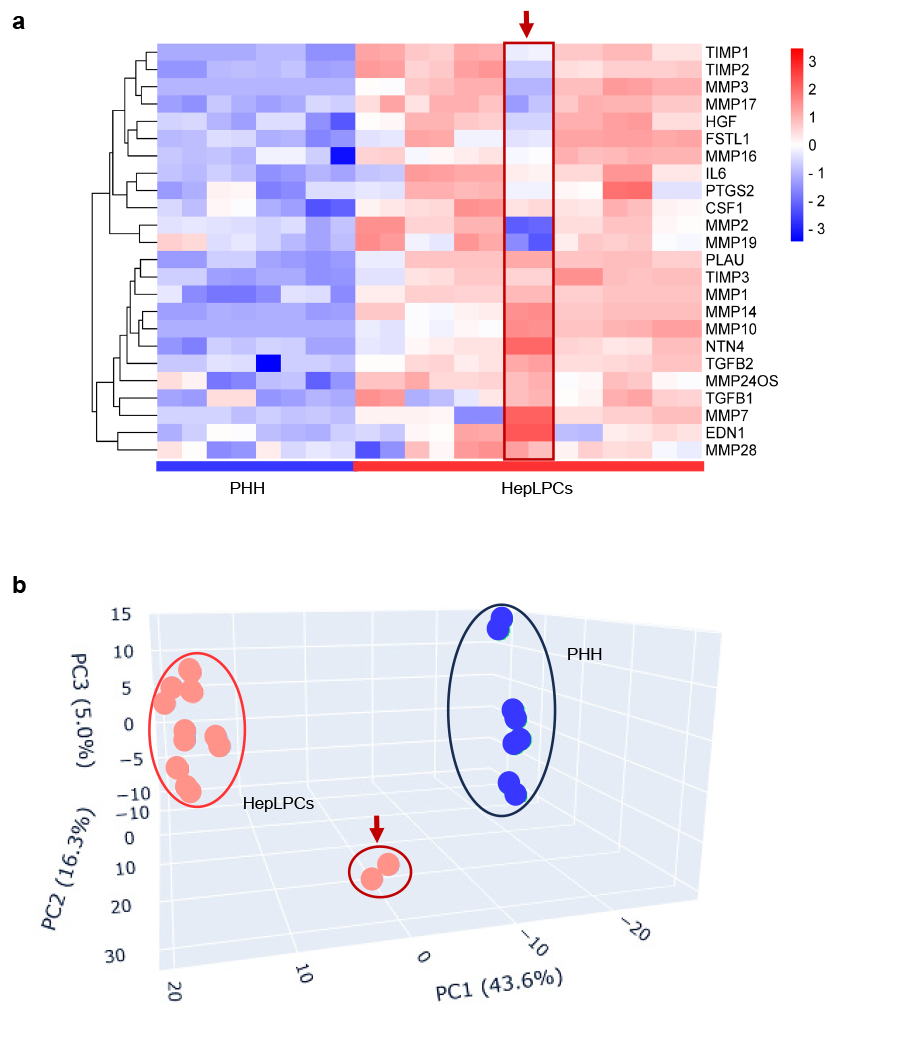
**

**Supplementary Figure 1.**

(a) Heat map showing the gene expression levels associated with regulation of extracellular matrix disassembly and hepatocyte growth factor receptor signaling pathway between primary hepatocytes from 4 donors and HepLPCs from 7 donors.

(b) Principal-component analysis (PCA) was performed to compare global gene expression profiles between Hepatocytes from 4 donors and HepLPCs from 7 donors. Each element represents the log2 (normalized expression), as scaled by the corresponding color legends. 2 independent experiments for each donor. Arrowhead, 1 out of 7 lines showing high variability.


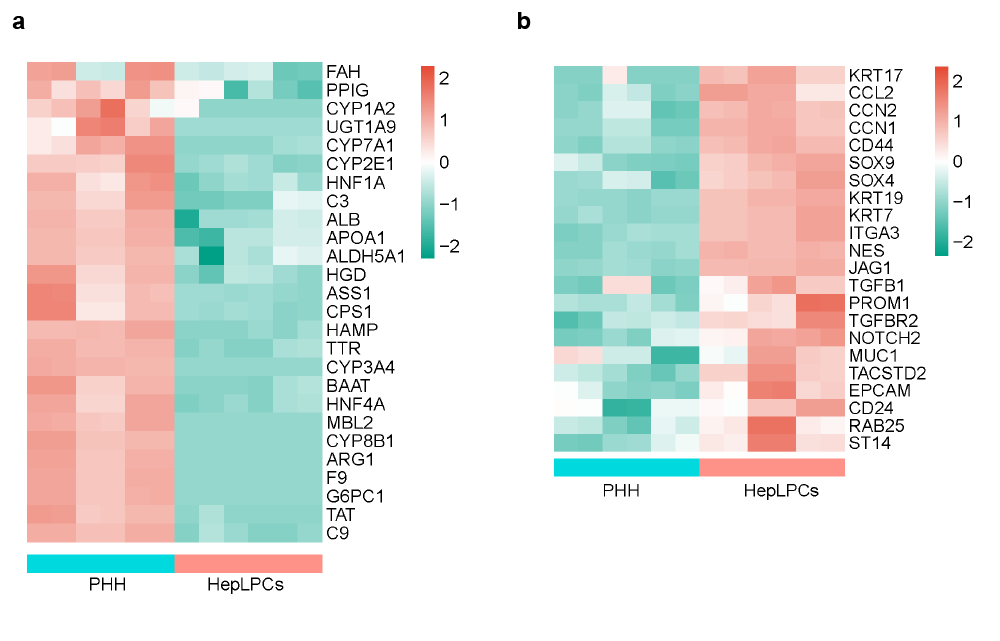


**Supplementary Figure 2.**

(a) Heat map showing the gene expression levels associated with general hepatocyte markers. n = 3 donors (2 independent experiments for each donor)

(b) Heat map showing the gene expression levels associated with biliary markers. n = 3 donors (2 independent experiments for each donor)

**
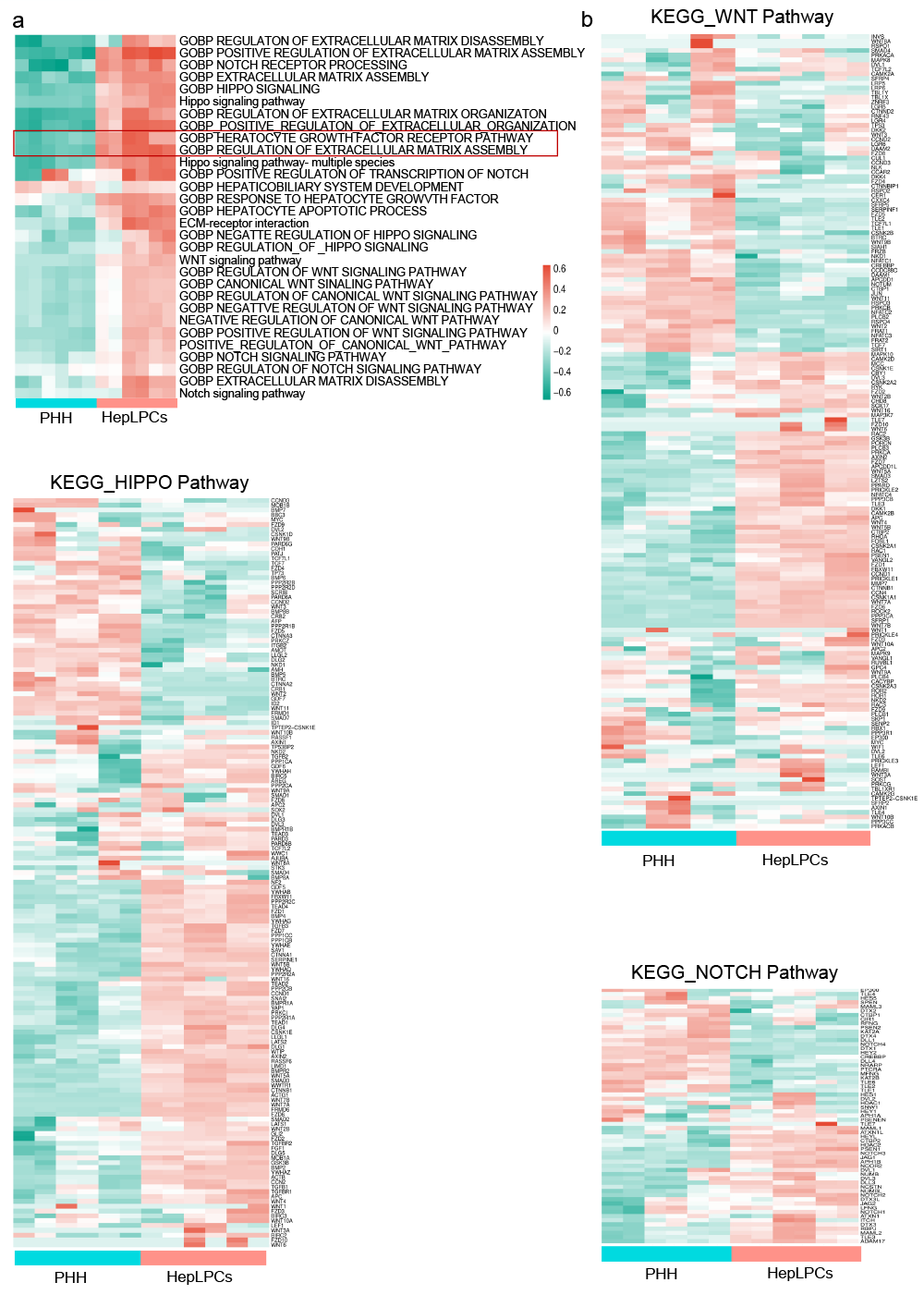
**

**Supplementary Figure 3.**

(a) Comparison of differential GO-BP and KEGG pathway activity by GSVA (gene set variation analysis). n = 3 donors (2 independent experiments for each donor).

(b) Heatmap showing the gene expression levels associated with WNT, NOTCH, and HIPPO signaling pathways.


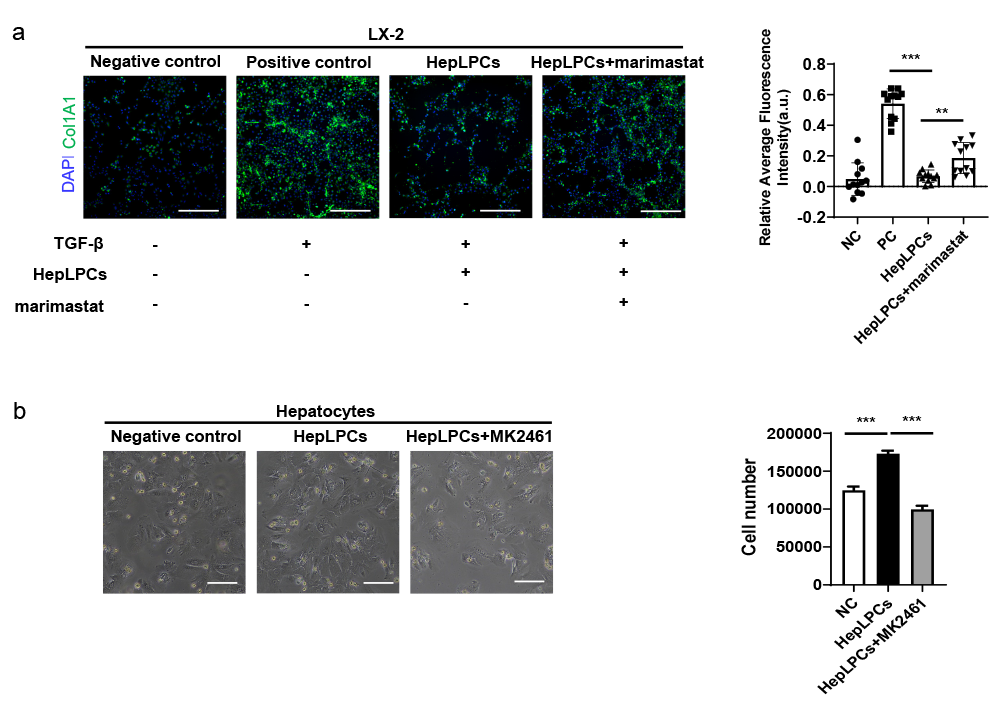


**Supplementary Figure 4.**

(a) The in vitro experiment used the human hepatic stellate cell line LX-2 as target cells, co-culturing the culture supernatant of HepLPCs with LX-2 (activated by TGF-β1) with or without MMPS inhibitors (marimastat, 20μM). Immunofluorescence was performed to label and quantify the fibrotic marker Collagen I, Scale bar = 100μm.

(b) The in vitro experiment used the human hepatocytes as target cells, co-culturing with the culture supernatant of HepLPCs with or without HGF/c-MET signaling antagonists (MK2461, 10μM). Scale bar = 50μm.

**
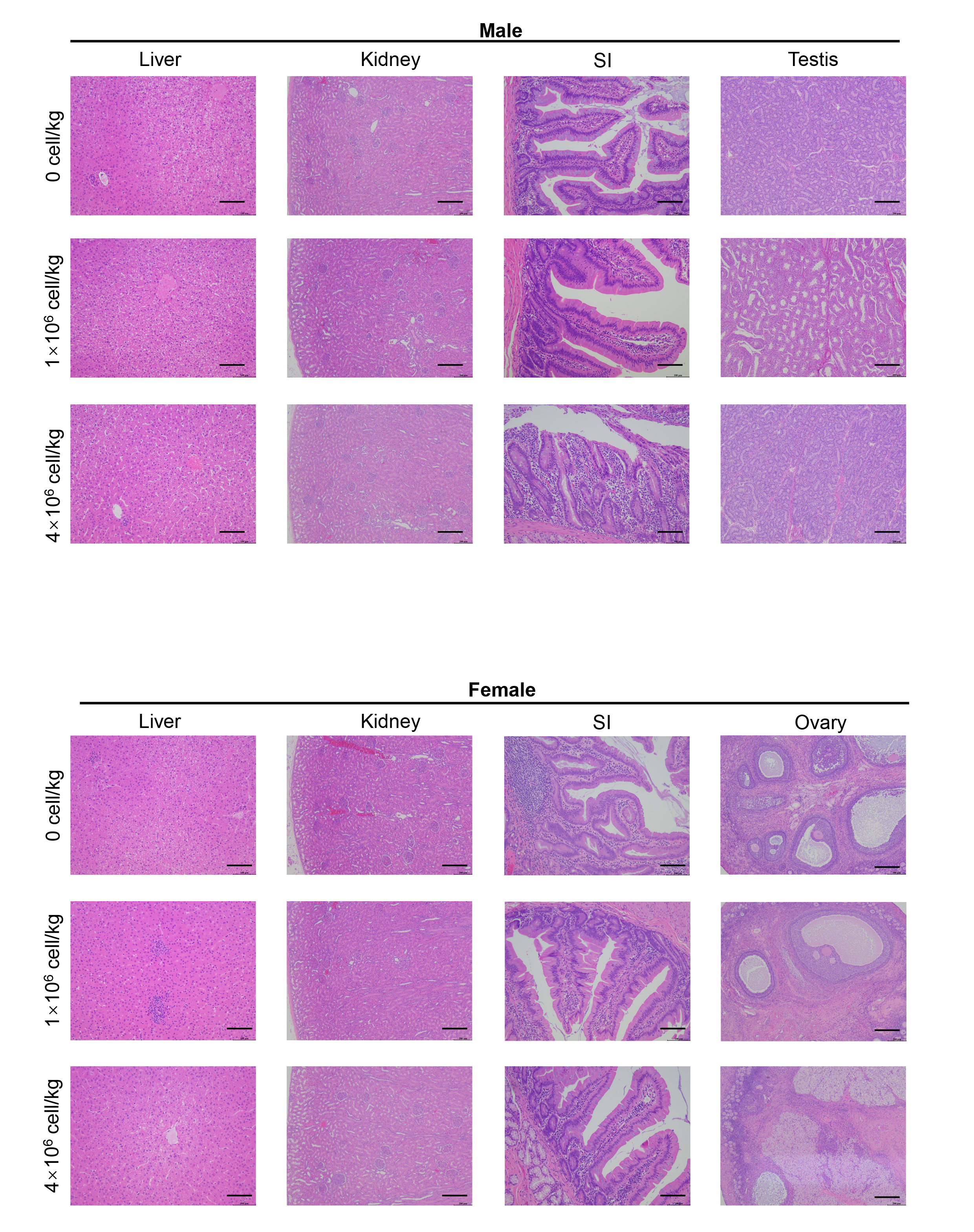
**

**Supplementary Figure 5.**

H&E staining of the organs collected from all groups after 5 weeks transplantation. Scale bars = 200 μm.


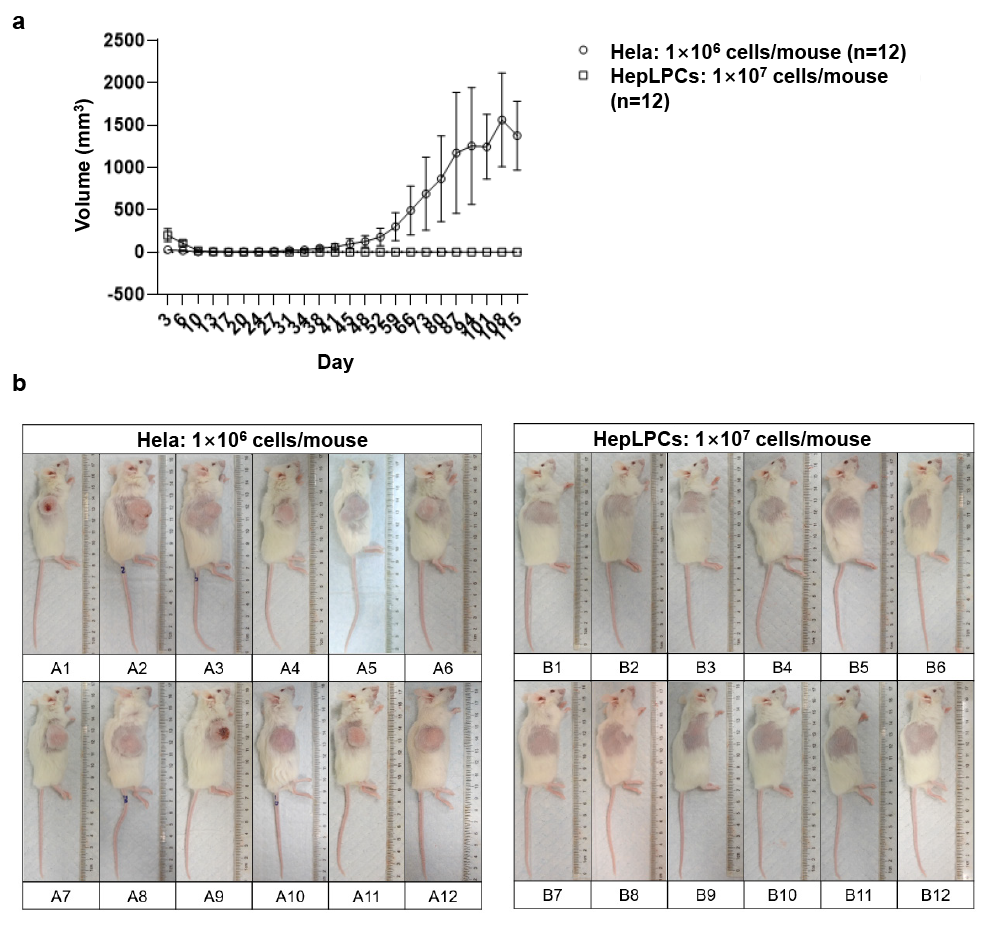


**Supplementary Figure 6.**

(a) Changes in tumor (or nodule) volume in each group of mice after cell inoculation.

(b) Tumor (or nodule) observation of mice in each group at the experimental endpoint.

**
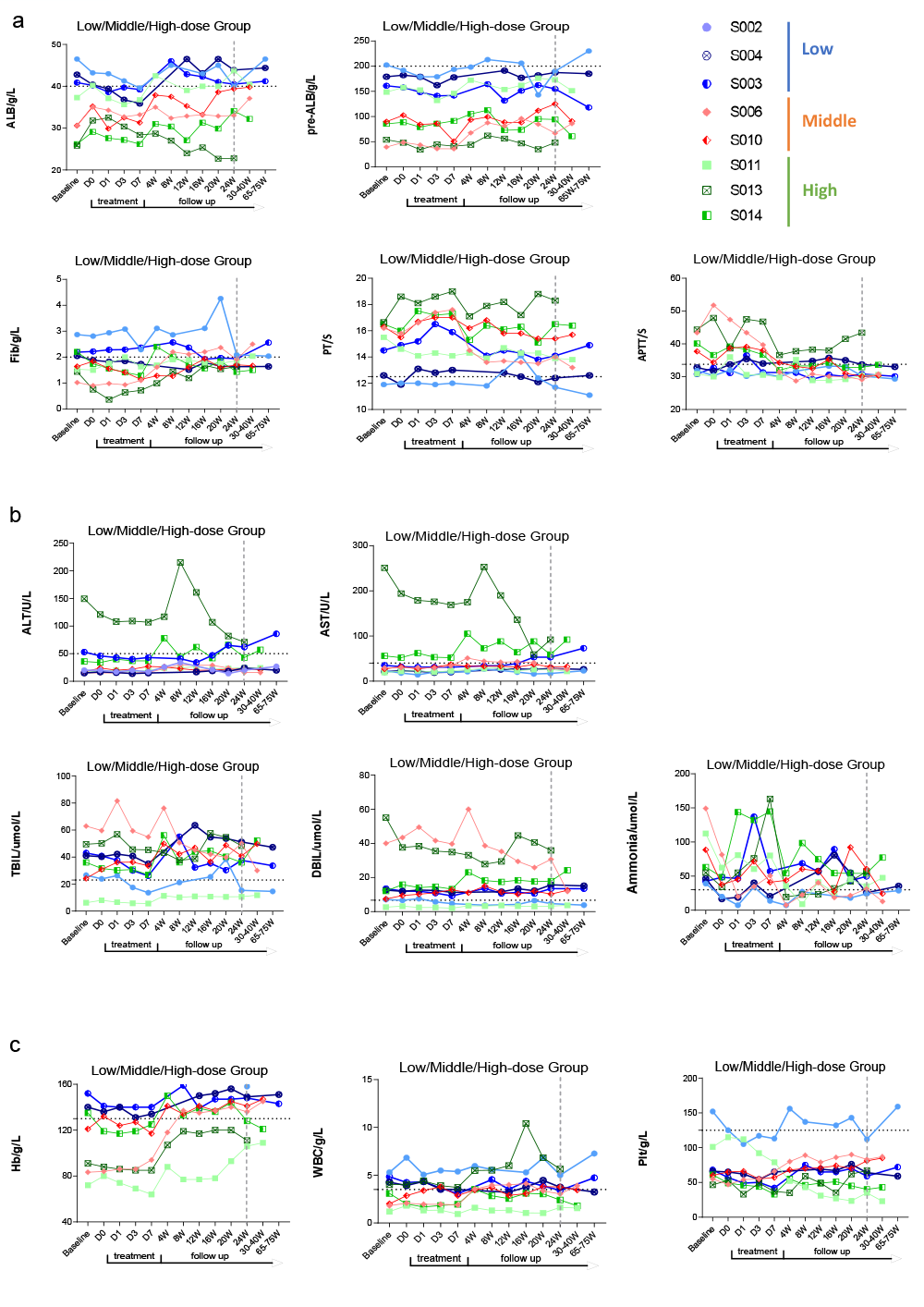
**

**Supplementary Figure 7.**

(a) Liver synthesis parameters, including Alb, Pre-Alb, Fib, PT and APTT, were detected in patients with cirrhosis before and after HepLPCs treatment.

(b) Liver function parameters, including ALT, AST, TBIL, DBIL and ammonia, were detected in patients with cirrhosis before and after HepLPCs treatment.

(c) Hematology parameters, including Hb, WBC and platelet, were detected in patients with cirrhosis before and after HepLPCs treatment.

Dashed lines correspond to normal values


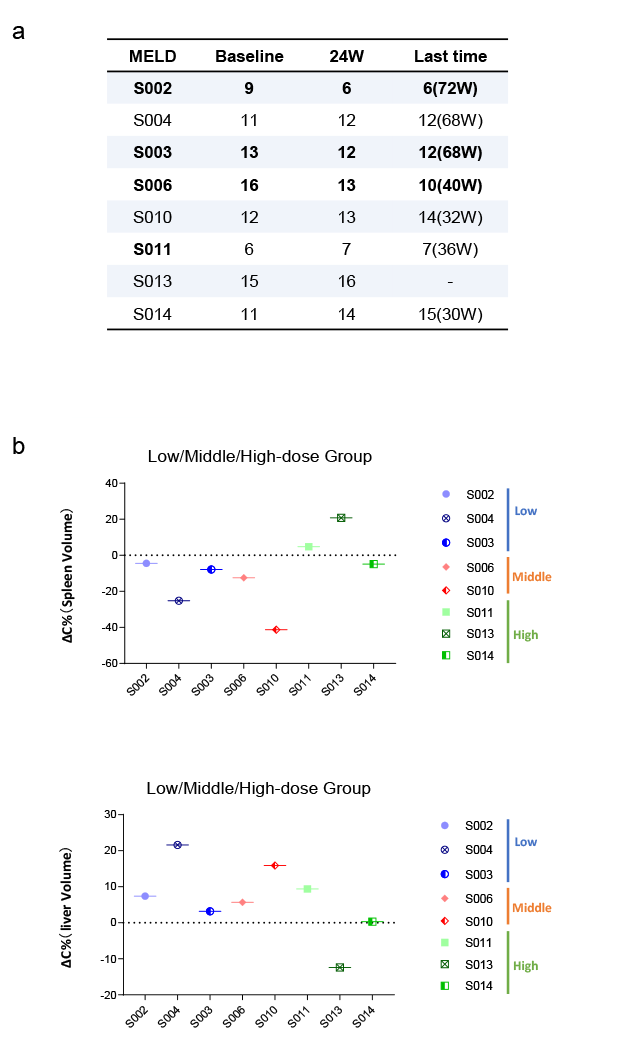


**Supplementary Figure 8.**

(a) The changes in MELD score in HepLPCs-treated patients.

(b) Spleen and liver volumes of patients with cirrhosis after HepLPCs treatment. ΔC = Value-Baseline, ΔC% = ΔC÷Baseline.


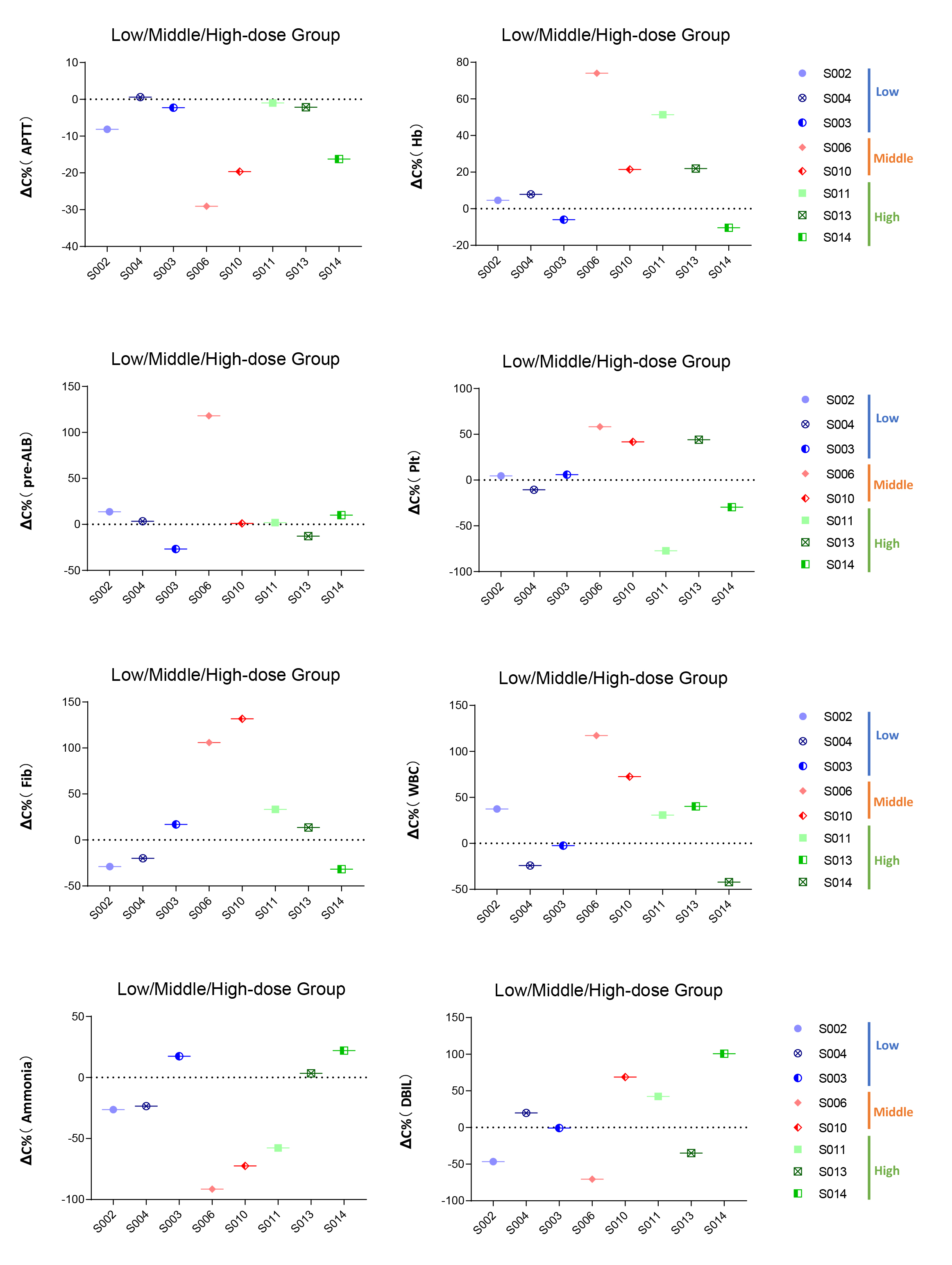


**Supplementary Figure 9.**

Liver synthesis parameters (Pre-Alb, Fib and APTT), Liver function parameters (DBIL and ammonia), and Hematology parameters (Hb, WBC and platelet), were detected in patients with cirrhosis before and after HepLPCs treatment.


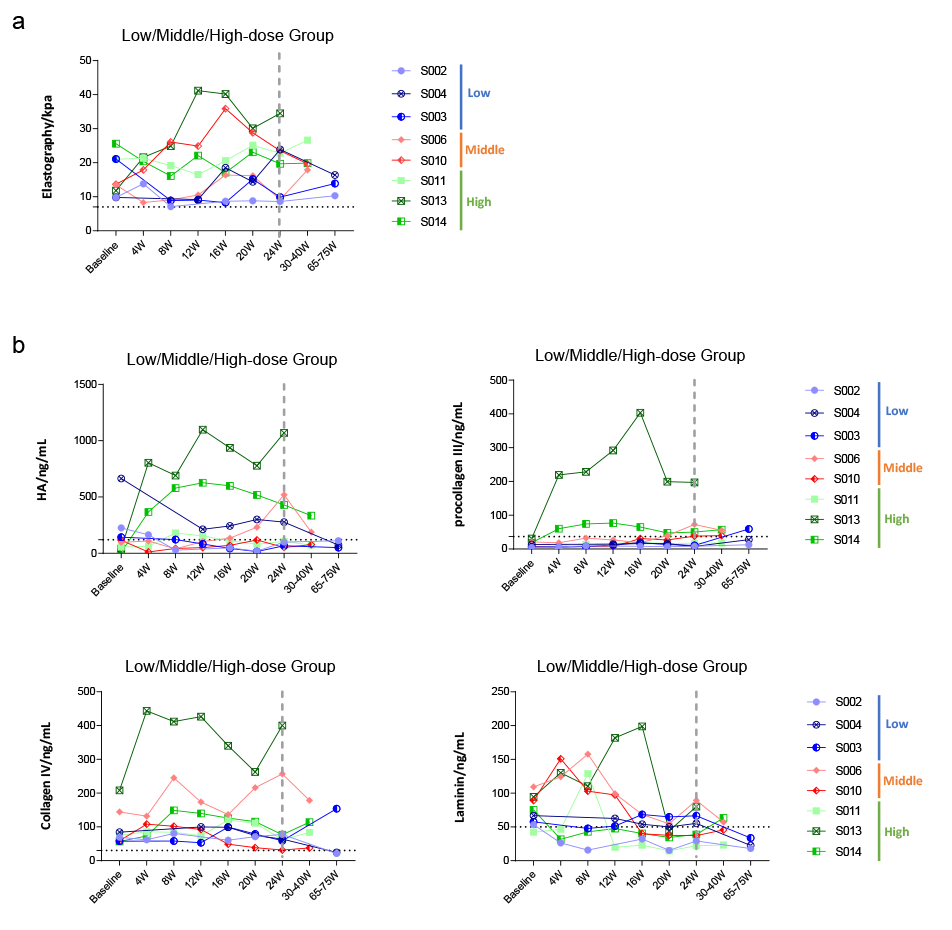


**Supplementary Figure 10.**

(a) Liver stiffness measurements (LSM) and (b) liver fibrosis markers (including HA, PC III, IV-C and LN) of patients with cirrhosis before and after HepLPCs treatment.

Dashed lines correspond to normal values


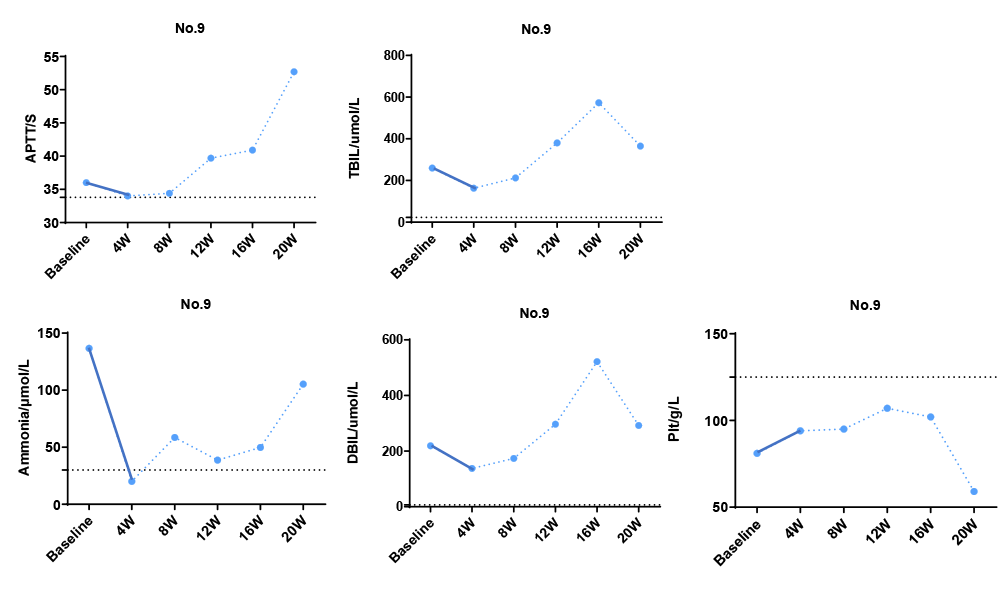


**Supplementary Figure 11.**

APTT, Ammonia, Plt, TBIL and DBIL were detected in patients No.9 before and after HepLPCs treatment.

**Supplementary Table 1. Baseline characteristics of trial participants classified by cell dose group**

| Table 1. Baseline characteristics of trial participants classified by cell dose group | | | | | | | | | | | | | | |
| --- | --- | --- | --- | --- | --- | --- | --- | --- | --- | --- | --- | --- | --- | --- |
| Participant ID  Demographics | Screen failure (n=5) | | | | | \| low dose (n=3) \| \| --- \| | | | \| medium dose (n=3) \| \| --- \| | | | \| high dose (n=3) \| \| --- \| | | |
|  | \| S001 \| \| --- \| | S005 | S007 | S008 | S012 | S002 | S003 | S004 | S006 | S009 | S010 | S011 | S013 | S014 |
| Gender | Male | Male | Male | Male | Male | Male | Male | Male | Male | Female | Female | Female | Female | Male |
| Age (years) | 54 | 51 | 51 | 55 | 60 | 37 | 41 | 60 | 58 | 53 | 63 | 59 | 51 | 59 |
| Body mass index (kgm-2) | 27.5 | 26.9 | 26.9 | 19.7 | 21.9 | 27.0 | 39.8 | 26.0 | 20.8 | 16.8 | 21.5 | 22.0 | 22.1 | 24.2 |
| Etiology of liver disease |  |  |  |  |  |  |  |  |  |  |  |  |  |  |
| HBV | ✓ | ✓ | ✓ | ✓ | ✓ | ✓ | ✓ | ✓ | ✓ |  | ✓ |  |  | ✓ |
| HCV |  |  |  |  |  |  |  |  |  |  |  |  | ✓ |  |
| PBC |  |  |  |  |  |  |  |  |  | ✓ |  | ✓ |  |  |
| Severity of cirrhosis |  |  |  |  |  |  |  |  |  |  |  |  |  |  |
| Meld score | 14 | 10 | 10 | 11 | 11 | 6 | 12 | 11 | 16 | 13 | 11 | 5 | 15 | 12 |
| Child-Pugh score | 7 | 5 | 5 | 7 | 5 | 5 | 5 | 6 | 9 | 9 | 7 | 6 | 8 | 8 |
| Child-Pugh class | B | A | A | B | A | A | A | A | B | B | B | A | B | B |

**Supplementary Table 2. Number of AEs during study periods**

| **Number of AEs during study periods** | | | | | |
| --- | --- | --- | --- | --- | --- |
| **AEs** | **Patients** | **infusion period + 7 days** | **up to 3 month** | **3-6 month** | **Total** |
| Flu-like symptoms | S002 |  |  | 1 | 1 |
| Sour throat | S003 |  | 1 |  | 1 |
| Right index finger contusion | S004 |  |  | 1 | 1 |
| Vommiting | S006 | 1 | 1 |  | 2 |
|  | S009 |  | 2 | 2 | 4 |
| Diarrhea | S006 |  | 1 |  | 1 |
|  | S011 | 1 |  |  | 1 |
|  | S013 |  |  | 1 | 1 |
| Nausea | S006 | 1 |  |  | 1 |
|  | S009 |  | 2 | 2 | 4 |
| Pruritus | S006 |  | 1 | 2 | 3 |
|  | S009 |  |  | 1 | 1 |
|  | S013 |  | 2 |  | 2 |
| SARS-CoV-2 infection | S006 |  |  | 1 | 1 |
|  | S009 |  |  | 1 | 1 |
|  | S010 |  |  | 1 | 1 |
|  | S011 |  | 1 |  | 1 |
| Chest discomfort | S006 |  |  | 1 | 1 |
| Urinary tract infection | S009 | 1 |  |  | 1 |
| Ecchymosis | S009 | 1 |  |  | 1 |
|  | S011 | 1 |  |  | 1 |
|  | S013 | 1 |  |  | 1 |
| Lower limb edema | S009 |  |  | 2 | 2 |
| Hypokalemia | S009 |  | 1 | 1 | 2 |
|  | S014 |  | 1 |  | 1 |
| Hyponatremia | S009 |  | 1 |  | 1 |
| SAE-Cholecystitis | S009 |  | 1 | 1 | 2 |
| Constipation | S009 |  |  | 2 | 2 |
|  | S013 | 0 | 4 |  | 4 |
| Cough | S009 |  |  | 1 | 1 |
| SAE-ACLF | S009 |  |  | 1 | 1 |
| Hepatic encephalopathy-phase II | S009 |  |  | 1 | 1 |
| Coagulation disorders | S009 |  |  | 1 | 1 |
| Microscopic hematuria | S009 |  |  | 1 | 1 |
| Hypoglycemia | S009 |  |  | 1 | 1 |
| Metabolic alkalosis | S009 |  |  | 1 | 1 |
| Upper limb edema | S010 |  | 2 |  | 2 |
| Gingivitis | S010 |  | 1 |  | 1 |
| Right thumb contusion | S010 |  | 1 |  | 1 |
| Hypertension | S010 |  | 1 |  | 1 |
| Lower limb spasm | S010 |  |  | 1 | 1 |
| Abdominal distension | S013 |  | 1 | 1 | 2 |
| **Total** |  | **7** | **25** | **29** | **61** |

**Supplementary Table 3. Visit Protocol Checklist**

| **Visit Procedure** | **Screening Period** | **Baseline** | **Treatment Period** | | | | **Follow-up Period** | | | | | |
| --- | --- | --- | --- | --- | --- | --- | --- | --- | --- | --- | --- | --- |
| **Study Visit（Day n）** | **V1** | **V2** | **V3** | | | | **V4** | **V5** | **V6** | **V7** | **V8** | **V9** |
|  | **D-30 to D0** | **D-7 to D0** | **D0** | **D1** | **D3** | **D7** | **W 4** | **W 8** | **W 12** | **W 16** | **W 20** | **W 24** |
| **Clinical Assessment** | | | | | | | | | | | | |
| Informed consent | × |  |  |  |  |  |  |  |  |  |  |  |
| Demographics | × |  |  |  |  |  |  |  |  |  |  |  |
| Medical history | × | × |  |  |  |  |  |  |  |  |  |  |
| Physical examination | × | × |  |  |  |  | × | × | × | × | × | × |
| Vital sign | × | × | × | × | × | × | × | × | × | × | × | × |
| Chest CT | × |  |  |  |  |  |  |  |  |  |  |  |
| Echocardiogram | × |  |  |  |  |  |  |  |  |  |  |  |
| Electrocardiogram | × | × |  |  |  |  |  |  |  |  |  | × |
| Esophagogastroscopy | × |  |  |  |  |  |  |  |  |  |  |  |
| Child-Pugh & MELD |  | × |  |  |  |  | × | × | × | × | × | × |
| ECOG & KPS | × |  |  |  |  |  |  |  |  |  |  | × |
| ECG monitoring for 24 hrs |  |  | × |  |  |  |  |  |  |  |  |  |
| **Clinical Laboratory Tests** | | | | | | | | | | | | |
| Serum virology test | × |  |  |  |  |  |  |  |  |  |  |  |
| HBV-DNA | × |  |  |  |  |  | × | × | × | × | × | × |
| HCV-RNA | × |  |  |  |  |  | × | × | × | × | × | × |
| AFP | × |  |  |  |  |  | × | × | × | × | × | × |
| Serum β-HCG | × | × |  |  |  |  |  |  |  |  |  | × |
| Complete Blood Count | × | × | × | × | × | × | × | × | × | × | × | × |
| liver and kidney function test, lipid profile, glucose, electrolytes, anmmoniaand CRP | × | × | × | × | × | × | × | × | × | × | × | × |
| HbA1c | × |  |  |  |  |  |  |  |  |  |  |  |
| Urinalysis | × | × | × | × | × | × | × | × | × | × | × | × |
| HLA | × |  |  |  |  |  |  |  |  |  |  |  |
| Interleukins & TNF-α |  | × |  |  |  |  | × |  | × |  |  | × |
| Complement |  | × |  |  |  |  | × |  | × |  |  | × |
| Immunoglobulin |  | × |  |  |  |  | × |  | × |  |  | × |
| Coagulation series | × | × | × | × | × | × | × | × | × | × | × | × |
| D-dimer | × | × | × | × | × | × | × | × | × | × | × | × |
| **Study treatment** | | | | | | | | | | | | |
| HepLPCs treatment (Day 0) |  |  | × |  |  |  |  |  |  |  |  |  |
| **Preliminary Efficacy Assessments** | | | | | | | | | | | | |
| Abdominal ultrasound | × |  |  |  |  |  | × | × | × | × | × | × |
| Hepatic vascular ultrasound & Elastography |  | × |  |  |  |  | × | × | × | × | × | × |
| Enhanced MRI | × |  |  |  |  |  |  |  |  |  |  | × |
| HA, PC III, IV-C and LN |  | × |  |  |  |  | × | × | × | × | × | × |
| Liver function test | × | × | × | × | × | × | × | × | × | × | × | × |
| **Safety Assessments** | | | | | | | | | | | | |
| Adverse events |  |  | × | × | × | × | × | × | × | × | × | × |
| Conconmitant medication | × | × | × | × | × | × | × | × | × | × | × | × |
